# A Framework for Sensor-Based Assessment of Upper-Limb Functioning in Hemiparesis

**DOI:** 10.3389/fnhum.2021.667509

**Published:** 2021-07-22

**Authors:** Ann David, Tanya Subash, S. K. M. Varadhan, Alejandro Melendez-Calderon, Sivakumar Balasubramanian

**Affiliations:** ^1^Department of Applied Mechanics, Indian Institute of Technology - Madras, Chennai, India; ^2^Department of Bioengineering, Christian Medical College, Vellore, India; ^3^Biomedical Engineering, School of Information Technology and Electrical Engineering, The University of Queensland, Brisbane, QLD, Australia

**Keywords:** wearable sensors, upper-limb rehabilitation, arm and hand use, sensorimotor assessment, real world activity, stroke rehabilitation, framework, hemiparesis

## Abstract

The ultimate goal of any upper-limb neurorehabilitation procedure is to improve upper-limb functioning in daily life. While clinic-based assessments provide an assessment of what a patient can do, they do not completely reflect what a patient does in his/her daily life. The use of compensatory strategies such as the use of the less affected upper-limb or excessive use of trunk in daily life is a common behavioral pattern seen in patients with hemiparesis. To this end, there has been an increasing interest in the use of wearable sensors to objectively assess upper-limb functioning. This paper presents a framework for assessing upper-limb functioning using sensors by providing: (a) a set of definitions of important constructs associated with upper-limb functioning; (b) different visualization methods for evaluating upper-limb functioning; and (c) two new measures for quantifying how much an upper-limb is used and the relative bias in their use. The demonstration of some of these components is presented using data collected from inertial measurement units from a previous study. The proposed framework can help guide the future technical and clinical work in this area to realize valid, objective, and robust tools for assessing upper-limb functioning. This will in turn drive the refinement and standardization of the assessment of upper-limb functioning.

## 1. Introduction

After neurological injury, individuals require physical rehabilitation to promote recovery, minimize disability, and maximize independent living. Despite years of research pointing to the benefits of repetitive practice, the time patients spend in inpatient rehabilitation settings is often much less than the recommended guidelines (De Wit et al., 2005; Barrett et al., 2018). Moreover, after discharge, patients do not have enough opportunities to do targeted movement therapy at home, sometimes leading to a pattern of “learned non-use” (André et al., 2004) and other compensatory strategies to accomplish daily activities.

Valid and reliable assessments are crucial for gaining a better understanding of a subject's sensorimotor state, and allowing us to tailor intervention strategies or improve health services. While clinic-based assessments of body function and activity can measure the capability of a patient, they are poor indicators of the actual use of a limb in day-to-day life (Mallinson and Hammel, 2010; Lemmens et al., 2012; Rand and Eng, 2012; Van Meulen et al., 2016). Thus, the assessment of movement behavior in natural settings is vital to evaluate recovery and the real-world impact of rehabilitation interventions. In the context of hemiparesis, such assessments can help gauge the extent to which changes in day-to-day activities can be attributed to true recovery or compensatory strategies.

There are four inter-related aspects that need consideration to build a comprehensive picture of upper-limb functioning in daily life: (a) amount of use (duration and/or intensity), (b) hand preference, (c) ability and capability, and (d) quality of movement. They can be posed as the following questions of interest to a clinician:

- **Q1**. How much is an upper-limb used during daily life?
- **Q2**. What is the relative preference for using the more-affected limb over the less-affected one?
- **Q3**. What kind of upper-limb tasks does the subject achieve in day-to-day activities?
- **Q4**. What is the quality of upper-limb movements performed during day-to-day activities?

Assessments such as the motor activity log (MAL) (Uswatte et al., 2006b) have been devised to capture, to an extent, such aspects of upper-limb functioning. In the MAL, the amount and quality of use are rated on a 11-point Likert scale for a set of pre-selected tasks. The amount and quality of the more-affected limb'use is reported by comparing it either to less-affected limb or to the pre-stroke condition of that limb. However, the MAL has limited sensitivity and relies on a patient's ability to recall upper-limb use from memory. Thus, the MAL can only provide a coarse and subjective evaluation of upper-limb functioning in daily life.

There is growing interest in wearable sensors for continuous and objective monitoring of upper-limb functioning (Bailey et al., 2014, 2015; McLeod et al., 2016; Bochniewicz et al., 2017; de Lucena et al., 2017; Lang et al., 2017; Leuenberger et al., 2017; David et al., 2020; Lum et al., 2020). Inertial sensors composed of accelerometers and gyroscopes have been the preferred modality for assessing upper-limb functioning in the natural setting, due to their availability, affordability, and compact size (Bailey et al., 2014, 2015; McLeod et al., 2016; Bochniewicz et al., 2017; de Lucena et al., 2017; Lang et al., 2017; Leuenberger et al., 2017; David et al., 2020; Lum et al., 2020). Thus far, the focus of sensor-based assessment in hemiparesis has been the quantification of the overall amount (Q1) and the relative bias (Q2) in using the upper-limbs during daily life (Bailey et al., 2014, 2015; McLeod et al., 2016; Bochniewicz et al., 2017; de Lucena et al., 2017; Lang et al., 2017; Leuenberger et al., 2017; David et al., 2020; Lum et al., 2020). The current methods for quantifying the amount of upper-limb use have either used: (a) the magnitude of acceleration [e.g., activity counting (AC) (Uswatte et al., 2006a; Bailey et al., 2014; de Lucena et al., 2017)] or (b) the duration of functional movements detected from sensor data [e.g., gross movement (GM) score (Leuenberger et al., 2017; David et al., 2020), machine learning (ML) algorithms (McLeod et al., 2016; Bochniewicz et al., 2017; Lum et al., 2020)]. Although related, movement duration and intensity convey slightly different information, and individually they only provide partial characterization of how much a particular arm is used. A complete picture of how much an arm is used in daily life requires knowledge of both the duration and the intensity of the upper-limb movements. Also, there is currently little work on using sensor data for determining the nature of tasks/activities and quantifying the quality of movements performed during daily life in neurorehabilitation application. These aspects are likely to be explored in the coming years with the increasing interest in this area, the availability of more data and sophisticated data analysis methods.

In order to develop rigorous methods to assess different aspects of upper-limb functioning, now is an opportune moment to lay a good foundation for this problem through a formal framework consisting of: (a) definitions of essential concepts and their interrelationships, (b) visualization methods for the information collected and computed from the sensor data, and (c) quantitative measures for different aspects of upper-limb functioning. Such a framework can help steer future technical developments in the appropriate direction, and limit work on ill-founded methods.

This paper presents a framework for the sensor-based assessment of upper-limb functioning, targeting researchers developing and validating quantitative methods for sensorimotor assessments. This framework focuses on questions Q1 and Q2 described earlier, which is necessary for answering the other two questions. The paper starts with formal definitions of the various concepts (section 3) of the framework. This is followed by qualitative and quantitative analysis (section 4) of different methods for visualizing how much an upper-limb is used (Q1), and the relative bias between the two upper-limbs (Q2). We note two important points about the current work to set the right context for the reader:

1. The work presented here is theoretical in nature concentrating primarily on clearly defining essential concepts and delineating their relationships. Demonstrations of the different concepts, new visualizations, and measures are preliminary in nature, carried out through data collected from a previous pilot clinical study (David et al., 2020).
2. The framework presented in sections 3–4 does not make any overt assumptions on the sensing modality used for assessing upper-limb functioning. The exact nature of the sensing modality can have a major influence on the fidelity of the assessment.

The clinical relevance of the proposed framework, followed by its limitations are discussed in the final section of the paper section 5. We also bring to light some important issues that should be addressed in the coming years for making pervasive, sensor-based objective assessment of upper-limb functioning a clinical reality.

## 2. The Nature of Assessment of Upper-Limb Functioning

**Figure 1:**
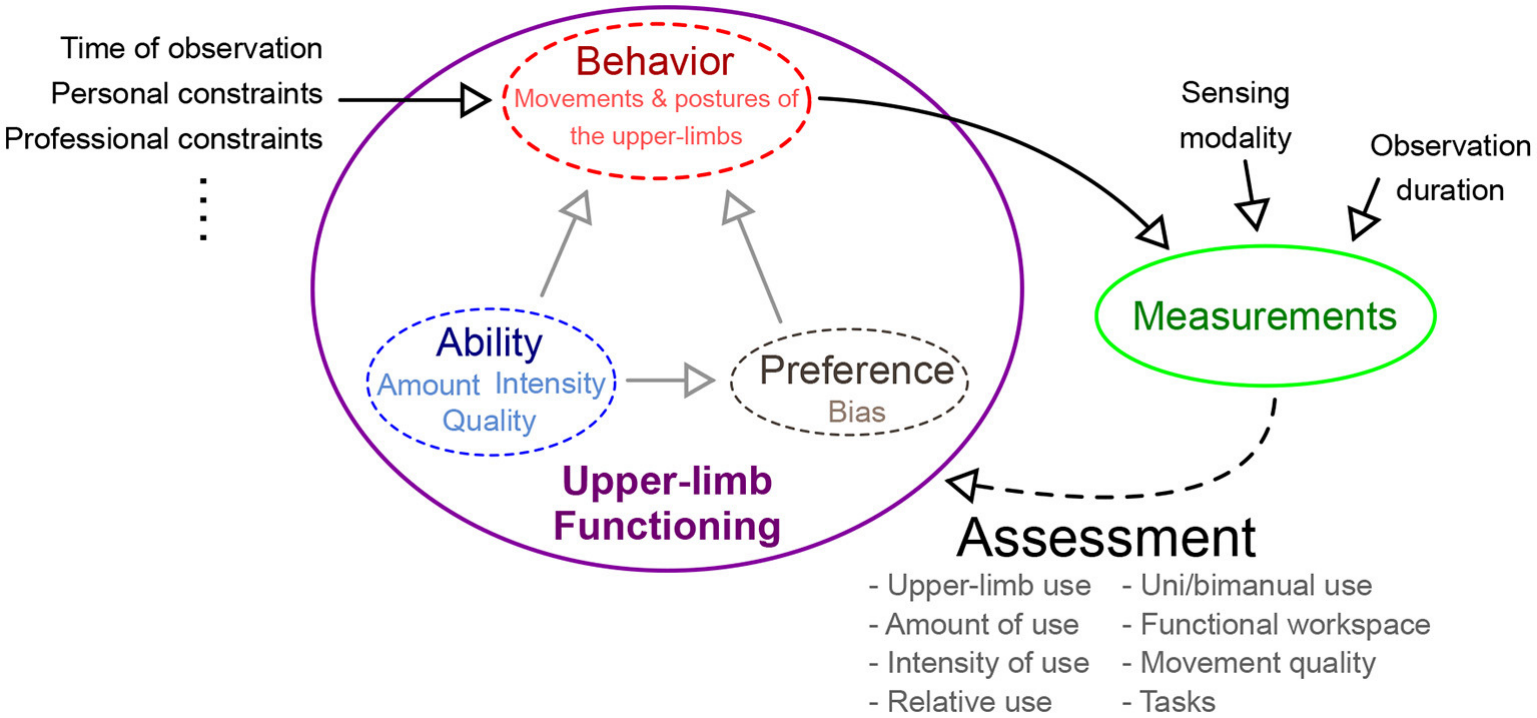
Different factors affecting the nature of use of the two upper-limbs during daily life. The construct of upper-limb functioning is composed of one's ability, preference, and observable behavior. Behavior is affected by multiple factors: *intrinsic* and *extrinsic* factors. Intrinsic factors inherent to a subject, e.g., ability and preference, while extrinsic factors are external to the subject. Measurements of behavior are used by an assessment procedure to estimate upper-limb functioning.

Any assessment of human behavior in a natural setting is a non-trivial undertaking due to the lack of control over important confounding factors influencing behavior (e.g., desk vs. manual jobs would lead to very different movement patterns), and the constraints in measuring the information of interest (e.g., privacy/security issues). Note that behavior in this context refers to the different tasks, postures, and movements carried out by a subject. In standard clinic-based assessments of motor ability (e.g., FMA, ARAT, etc.), these factors are controlled by defining them as part of the assessment protocol (e.g., definition of the task, limb to be used, measurement approach etc.). A controlled environment for assessment enables clear interpretation of the observed movement behavior, and simplifies intra- and inter-subject comparison of motor abilities. This luxury is absent in the assessment of upper-limb functioning in natural settings, which uses measurements during unconstrained behavior to assess the different aspects of upper-limb functioning (questions Q1–Q4 in section 1). Behavior is affected by two types of factors:

- *intrinsic factors* that are directly related to upper-limb functioning, which are to be estimated by the assessment process (shown within the purple ellipse in Figure 1). The motor ability and the preference for the two upper-limbs will determine the types of tasks, amount, and quality of movements performed by a subject during daily life. For example, lower ability is likely to reduce the overall use of the affected upper-limb and result in poorer quality of movements. This will also discourage its use in complex, high intensity, and long duration tasks. Similarly, a subject might avoid doing fine manipulation tasks with the affected dominant upper-limb.
- *extrinsic factors* are confounders that influence behavior and thus affect assessment outcome (listed on the left of the purple ellipse in Figure 1). Some of these factors include the time of observation of behavior, personal and professional constraints, etc. For instance, the observed behavior is likely to be different earlier vs. latter in a day, or the day of the week/month etc., due to changes in requirements and constraints of daily routine. Similarly, constraints from personal and professional life will influence behavior.

Interpretation of behavior through measurements is influenced by two factors, namely, the nature of the sensing modality used, and the duration for which a subject is observed (shown above the green ellipse in Figure 1). The exact choice of sensing modality is constrained by conflicting requirements of being minimally obtrusive while gathering maximal information for the accurate assessment of upper-limb functioning. An ideal sensing modality must be compact, wearable, aesthetic, and must comply with the necessary privacy requirements, while still gathering rich behavioral information. Furthermore, the duration of observation will also determine the quality of information gathered for assessment; longer observation periods are likely to better capture “typical” behavior than shorter ones, and thus provide a less biased estimate of different aspects of upper-limb functioning. However, longer observations periods are likely to have poor compliance, due to inconvenience in using the sensors for recording daily behavior.

Thus, the assessment of upper-limb functioning must control for as many extrinsic factors as possible (e.g., fix the time of observation within and across subjects), while also ensuring the choice of sensing modality and the duration of observation are kept unchanged. This can minimize the effect on assessment outcome variability within and across subjects, thus improving the interpretability of the outcomes. It is, however, crucial to be aware that there are other extrinsic factors that still influence the outcome and thus interpreting outcomes of upper-limb functioning must be done with care.

## 3. Assessing Upper-Limb Functioning: Formal Definitions

Before getting into the details of the framework, we start with a brief overview of the general process of sensorimotor assessment in the context of neurorehabilitation. This detour is necessary to establish the meanings of the terms “evaluation,” “assessment,” “measure,” and “measurements,” as some of these terms are used to mean different things in the current literature. The process of determining the sensorimotor state of a subject is a hierarchical process with clinical evaluation at its highest level. We define evaluation as the process of interpreting the results of one or more assessments to gauge a subject's sensorimotor state with respect to a reference, either him/herself from a different time point (intra-subject), or another subject (inter-subject). For instance, an evaluation is performed when comparing the results of ARAT assessments across different time points, or comparing smoothness of reaching movements of a patient against normative data. Evaluations can be aided through visualizations that allow interpretation of assessments. One level below in this hierarchy are assessments, which we define as the process of quantifying (i.e., putting numbers) abstract theoretical constructs (e.g., smoothness, coordination, synergies). For instance, the Fugl-Meyer upper-limb assessment is a process of quantifying the constructs “motor function,” “synergy,” and “coordination.” Unlike an evaluation, an assessment only deals with assigning numbers (or labels in some cases) to constructs of interest. Assessments require clearly defined protocols for collecting data (e.g., tasks/movements to be performed), and measures. A measure is a well-defined mathematical function/formula, a computational algorithm, or a set of rules for mapping measurements or observations to quantities that are interpretable in the context of the given construct. For instance, spectral arc length (SPARC) and log dimensionless jerk (LDLJ) are measures of the construct “movement smoothness”; the rules used for assigning a score to the flexion synergy task in the Fugl-Meyer assessment is a measure of the construct “flexion synergy.” Measures with good properties are essential to obtain valid, reliable, and interpretable assessments. Finally, measurements are records of variables (e.g., speed, position, orientation, etc.) obtained through various sensors or through human observation. Measurements are used by measures to quantify constructs, e.g., measurements of movement speed profile are used by the SPARC measure to quantify movement smoothness.

**Figure 2:**
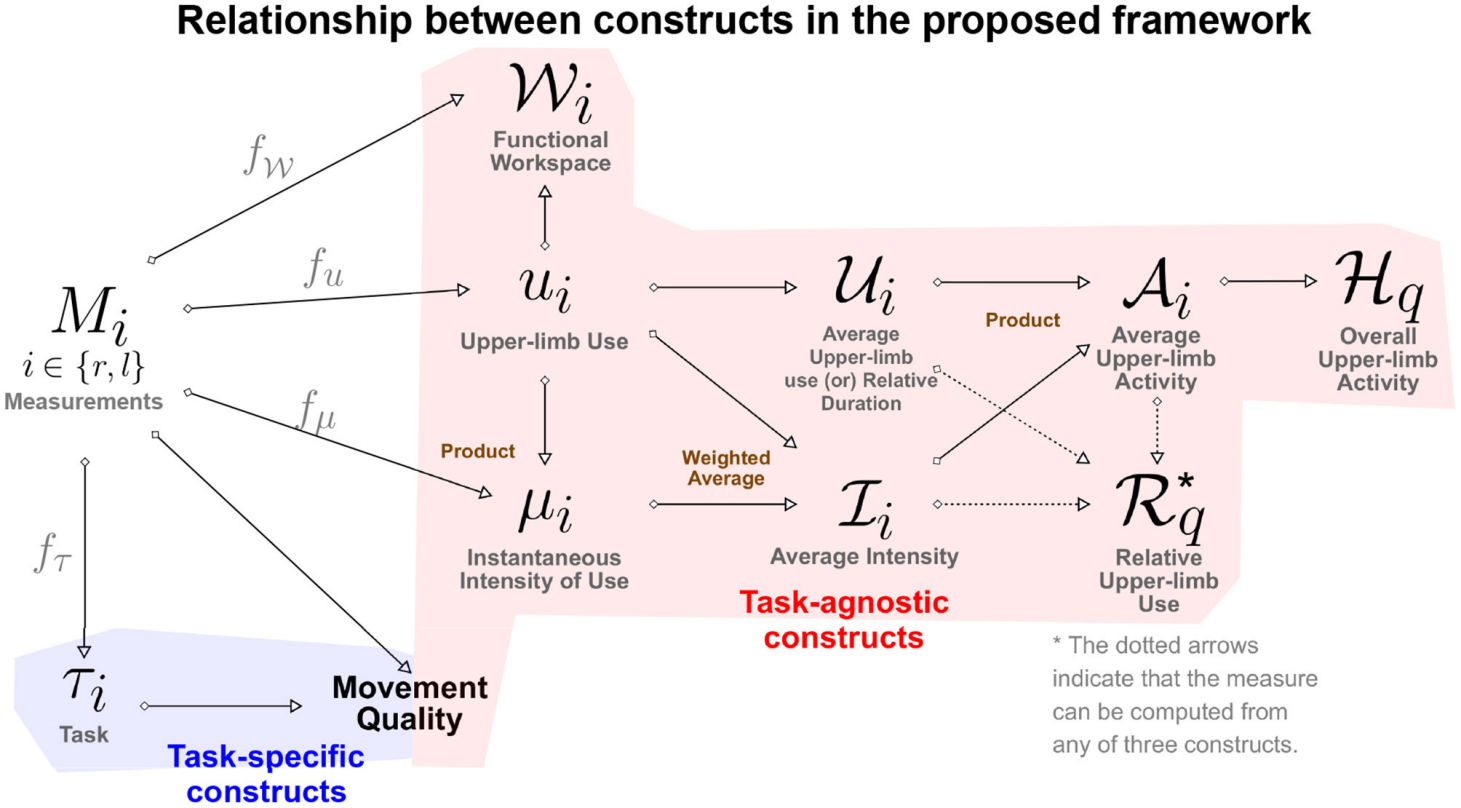
A directed graph representation of the connections between the different constructs defined in the proposed framework. The leftmost node represents the measurements, while the rest of the nodes are constructs of interest in the assessment of upper-limb functioning. The construct at the end of a directed edge is derived using the construct/measurements at the start of the directed edge. The measures (gray color text) used to quantify a construct from measurements are placed above the directed edge. The brown colored text next to some of the construct indicate how two constructs are combined to derive the target construct.

In the rest of this section we provide definitions of the constructs of the proposed framework for assessing upper-limb functioning. The relationship between various constructs introduced is in this section is summarized in Figure 2. Relevant literature that supports the constructs defined below are given in Table 1. We make no explicit assumption on the types of measurements available for quantifying the different constructs defined below. It should, however, be evident that the different types of measurements will vary in terms the amount of information they convey about the different‘constructs.

**Table 1:** List of studies that have implemented measures related to upper limb functioning during free living conditions.

**Construct**	**Literature**
Measurement space (*M*_*i*_)	Wrist-worn Accelerometers (Uswatte et al., 2006a; Bailey et al., 2014, 2015) Wrist-worn Inertial measurement units (Bochniewicz et al., 2017; de Lucena et al., 2017; David et al., 2020; Lum et al., 2020) Finger-worn magnetic ring (Friedman et al., 2014) Egocentric camera (Nguyen et al., 2016; Tsai et al., 2020, 2021)
Upper-limb use (*u*_*i*_)	Thresholded activity count (Lum et al., 2020; Subash et al., 2020) Gross movement score algorithm (Leuenberger et al., 2017; David et al., 2020) Random forests algorithm (Bochniewicz et al., 2017; Lum et al., 2020)
Instantaneous intensity of use (μ_*i*_)	Activity count (Uswatte et al., 2006a; Bailey et al., 2014, 2015; de Lucena et al., 2017)
Average upper-limb use $\left(U_{i}\right)$	Mean arm-use (David et al., 2020)
Average intensity of use $\left(I_{i}\right)$	None^*^
Average upper-limb activity $\left(A_{i}\right)$	None^*^
Task (τ_*i*_)	for detecting tasks using different sensors (e.g., Bulling et al., 2014; Nweke et al., 2018)
Movement quality	None^*^

*The components of the framework that have not been explored in the current literature, entry is filled as “None”*.

* *means that this is to best of the authors' knowledge*.

### 3.1. Measurement Space

The type of sensor measurements available for an assessment will determine the steps in the analysis pipeline and the nature of information extracted about a subject's sensorimotor state. It is thus crucial for any assessment procedure to clearly state the measurement variables used to quantify a specific construct. In this context, we define measurement space as the following.

*Definition* Measurement space is the universal set of all possible sensor measurements available from an upper-limb for quantifying the different constructs in an assessment.

We denote this set—the measurement space—by 𝕄 and assume that the same quantities are measured from both upper-limbs for the given assessment procedure. Inertial sensing is one of the most common modalities used in the current literature, where the arm movements are measured using wrist-worn accelerometers (𝕄 = ℝ^3^) or IMUs^1^ (𝕄 = ℝ^6^). For a more elaborate measurement setup that includes wrist position and orientation, along with *k* joint angles of the arm, 𝕄 = ℝ^3^ × *SO*(3) × [0, 2π)^*k*^; where ℝ^3^ is the set of all possible wrist positions, *SO*(3) is the special orthogonal group of all rotation matrices representing 3D orientations of the wrist, and [0, 2π)^*k*^ is the set of *k* joint angles.

Measurements for an assessment are made over a finite observation period referred to as the *measurement epoch*. Let *M*_*l*_(*t*) and *M*_*r*_(*t*) represent the values of the measurements from the left and right upper-limb, respectively, made at time instant *t*, where *t* ∈ [0, *T*], and *M*_*l*_(*t*), *M*_*r*_(*t*) ∈ 𝕄; *T* is the duration of the measurement epoch. We use *M*_*l*_ and *M*_*r*_ to represent the entire time series or signal, where *M*_*l*_, *M*_*r*_ ∈ 𝕄([0, *T*]); 𝕄([0, *T*]) is the set of all possible measurement signals over a measurement epoch of duration *T* seconds starting at time *t* = 0. In addition to specifying 𝕄, it is also essential for the reproducibility of an assessment to clearly specify the exact sensors used for the measurements, their accuracy, noise characteristics, resolution, sampling rate, etc. The values of these parameters have practical implications for data analysis and interpretation. These practical issues will be not be considered in this manuscript, and to simplify the presentation the mathematical formalism used assumes all measurements to be continuous in time and space.

### 3.2. Upper-Limb Use

*Definition* Upper-limb use is a binary construct indicating the presence or absence of a voluntary, meaningful movement, or posture of an upper-limb.

In this definition, the boundary of what constitutes a “meaningful” movement/posture must be defined a priori. Some examples of meaningful use include reaching and grasping, turning a doorknob, stabilizing an object with one limb while manipulating it with the other, holding a glass, writing, typing, upper-limb therapy exercises etc. Under this definition, involuntary and passive upper-limb movements/postures are not considered meaningful, e.g., resting the arm on a table, upper-limb moved by an external force, etc. There are, however, cases where the presence/absence of upper-limb use is ambiguous, e.g., arm swing during walking, passively resting the upper-limb on a book to prevent the pages from turning, etc. Such ambiguities are best resolved in an application-specific manner, where the set of tasks considered as meaningful are clearly stated a priori. For instance, in the current upper-limb use literature arm swing during walking is not considered as meaningful, even though these are unlikely to be purely passive movements (Blouin and Fitzpatrick, 2010).

Upper-limb use can be mathematically represented as a binary signal over time computed from upper-limb measurements *M*_*i*_ ∈ 𝕄([0, *T*]), where *i* ∈ {*l, r*}. Let *f*_*u*_ be a function representing a measure that maps a given measurement signal *M*_*i*_ to a binary signal *u*_*i*_ over the same temporal domain, i.e., *f*_*u*_:𝕄([0, *T*]) ↦ 𝔹([0, *T*]); 𝔹([0, *T*]) is set of Riemann-integrable binary signals in the time interval [0, *T*].

(1) $$u_{i}≜f_{u}\left(M_{i}\right),\;u_{i}\left(t\right)≜\{\begin{array}{ll} 0, & \text{UL is not in use at time }t\\ 1, & \text{UL is in use at time }t \end{array}$$

where, *u*_*i*_ is the upper-limb use signal of the upper-limb *i*. The choice of *f*_*u*_ is determined by several factors, such as the measurement space 𝕄, computational complexity of the measure, sensitivity/specificity of the measure, etc. All measures of upper-limb use exploit some common structure in functional/meaningful movements present in the measured data to detect upper-limb use. Some examples of the current measures (*f*_*u*_) that make use of accelerometers or IMUs are:

- **Thresholded activity counting**. Activity counting (AC) is one of the most popular methods in the literature to quantify upper-limb functional and non-functional activity (Uswatte et al., 2006a; Bailey et al., 2014, 2015; de Lucena et al., 2017). AC has high sensitivity, but poor specificity (Subash et al., 2020). AC can be used with both accelerometers and IMUs. Upper-limb use *u*_*i*_ is computed from AC by assigning a value of 1, whenever the AC is above a threshold.
- **Gross movement (GM) score**. The Gross movement (GM) score (a.k.a Gross Counts or Gross Movement Identification method) proposed by Leuenberger et al. (2017) reconstructs the forearm orientation using a wrist-worn IMU to detect movements that occur in a pre-specified range of forearm orientations (Leuenberger et al., 2017). It is 1 whenever there are arm movements in a pre-specified range of forearm orientations, else it is 0. The GM score is highly specific, but has low sensitivity (Subash et al., 2020), and it can only be used with an IMU.
- **Random forests classifier**. Bochniewicz et al. (2017) proposed the use of a random forests classifier to detect upper-limb use from features extracted from an accelerometer. The ML approach can be used with both accelerometers and IMUs, and has reasonable sensitivity and specificity (Lum et al., 2020).

Upper-limb use as defined in this section is an idealized construct, and its detection in practice using sensor measurements will be error-prone due to measurement noise, the natural intra- and inter-subject movement variability, and the relative sensitivity of the sensor measurements to movements and postures. The nature of the measurements 𝕄 and the choice of measure *f*_*u*_ will influence how well upper-limb use can be quantified (e.g., sensitivity and specificity) in practice.

The upper-limb use signals from the two limbs can be used for defining unimanual and bimanual upper-limb use at time *t* as the following:

(2) $$\begin{array}{l} \begin{array}{cc} \text{Unimanual use of the right limb:} & u_{r}\left(t\right)\cdot \left(1-u_{l}\left(t\right)\right)\\ \text{ Unimanual use of the left limb:} & u_{l}\left(t\right)\cdot \left(1-u_{r}\left(t\right)\right)\\ \text{ Bimanual use of both limbs:} & u_{r}\left(t\right)\cdot u_{l}\left(t\right) \end{array} \end{array}$$

Unimanual use refers to a situation where only one of the upper-limbs is used, i.e., only *u*_*r*_(*t*) or *u*_*l*_(*t*) is 1 at time instant *t*, but not both. On the other hand, bimanual use involves the use of both limbs simultaneously, i.e., both *u*_*r*_(*t*) and *u*_*l*_(*t*) are 1 at a given time instant *t*. Bimanual use will include a wide range of coordination patterns between the two limbs (Kantak et al., 2017), including completely independent use of the two limb (e.g., writing with one hand while holding and drinking from a cup with the other hand) to movement of the two limbs with tight spatio-temporal synchronization (e.g., holding and balancing a tray of glasses with both hands).

### 3.3. Instantaneous Intensity of Use

*Definition* Instantaneous intensity of use is a construct that reflects how strenuous a movement/posture is at a particular instant of time, when the upper-limb is in use.

Some examples of measures (*f*_μ_) to quantify instantaneous intensity of use include the magnitude of movement velocity, acceleration, interaction force, muscle activity, etc. Let μ_*i*_ represent the instantaneous intensity of use signal for the upper-limb *i*. It assumes non-negative values when the upper-limb is used, and is defined to be zero otherwise.

(3) $$\begin{array}{l} \mu_{i}≜u_{i}\cdot f_{\mu }\left(M_{i}\right) \end{array}$$

where, μ_*i*_ ∈ ℝ_≥0_([0, *T*]), and the function *f*_μ_:𝕄([0, *T*]) ↦ ℝ_≥0_([0, *T*]) computes instantaneous intensity of use signal from the upper-limb measurement signal *M*_*i*_.

The exact choice for *f*_μ_ is application-specific and dictated by *M*. We also note that results obtained from different types of measurements and different measures *f*_μ_ might not be comparable, e.g., the magnitude of movement velocity can be independent of the magnitude of movement acceleration. Thus, it is imperative to report the exact *f*_μ_ and its units when reporting the instantaneous intensity of use. Activity counting, as defined in Bailey et al. (2014), Bailey et al. (2015), and de Lucena et al. (2017), is an example of an instantaneous intensity of use measure in the current literature.

In general, μ_*i*_(*t*) will not be uniformly zero in a continuous interval of time *t* ∈ [*t*_1_, *t*_2_] where there is a functional movement. However, μ_*i*_(*t*) can be uniformly zero in a continuous interval under two circumstances:

1. *u*_*i*_(*t*) = 0, ∀*t* ∈ [*t*_1_, *t*_2_]: When there is no upper-limb use during this interval.
2. *f*_μ_(*M*_*i*_)(*t*) = 0, *t* ∈ [*t*_1_, *t*_2_]: When the upper-limb is used in a meaningful posture, *f*_μ_(*M*_*i*_)(*t*) can be uniformly zero in the interval *t* ∈ [*t*_1_, *t*_2_] for some choice of measurement signal and *f*_μ_. For example, activity count, magnitude of movement acceleration/velocity will be zero during an upper-limb posture. On the other hand, the magnitude of muscle activity controlling the upper-limb will not be zero even while holding a voluntary posture.

### 3.4. Average Upper-Limb Use or Relative Duration

*Definition* Average upper-limb use or relative duration is a construct that reflects the proportion of time an upper-limb is used in a given time period *D*.

Average upper-limb use at time *t*, denoted by $U_{i}\left(t;D\right)$, can be computed as the average value of *u*_*i*_ in the past *D* seconds.

(4) $$\begin{array}{l} U_{i}\left(t;D\right)≜\frac{1}{D}\int_{t-D}^{t}u_{i}\left(x\right)dx,\;t\in \left[D,T\right] \end{array}$$

$U_{i}\left(t;D\right)$ is a smoothed version of *u*_*i*_. We will drop *D* in the parenthesis in the rest of the manuscript and use it only if it needs to be explicitly mentioned. From Equation (4), we can immediately identify some essential properties of $U_{i}$:

- $U_{i}$ is a continuous-valued signal that can take any value in the closed interval [0, 1].
- The value of $U_{i}\left(t\right)$ indicates the proportion of time in the interval (*t* − *D, t*] where the upper-limb was used, i.e., *u*_*i*_(*t*) was 1. Thus, there are infinitely many *u*_*i*_s that can result in the same $U_{i}$.
- The value of the parameter *D* will depend on the application, and controls the amount of smoothing of *u*_*i*_; larger values of *D* will results in smoother $U_{i}$ while compromising time localization of the information conveyed by $U_{i}$. When *D* = *T*, then $U_{i}$ measures the proportion of time the upper-limb *i* was used over the entire measurement epoch.

### 3.5. Average Intensity of Use

*Definition* Average intensity of use is a construct that reflects the average intensity of movements during upper-limb use in a given time period *D*.

Average intensity of use $I_{i}\left(t\right)$ can be computed from the upper-limb use signal *u*_*i*_ and the instantaneous intensity of use signal μ_*i*_(*t*) as the following,

(5) $$I_{i}\left(t;D\right)≜\{\begin{array}{ll} \frac{\int_{t-D}^{t}\mu_{i}\left(x\right)dx}{\int_{t-D}^{t}u_{i}\left(x\right)dx}, & \int_{t-D}^{t}u_{i}\left(x\right)dx\neq 0\\ 0, & \int_{t-D}^{t}u_{i}\left(x\right)dx=0 \end{array}$$

where, $I_{i}\left(t\right)\in {\mathbb{R}}_{\geq 0}$. The same ambiguity as μ_*i*_(*t*) exists when $I_{i}\left(t\right)=0$ for some time instant *t*. $I_{i}\left(t\right)=0$ could mean that the upper-limb was either not used during the time interval (*t* − *D, t*] or it was used for performing upper-limb postures, depending on the measure used to quantify instantaneous intensity of use.

### 3.6. Average Upper-Limb Activity

The amount of use of an upper-limb during a measurement epoch depends on both the duration and intensity of movements performed during this period, which are captured by $U_{i}$ and $I_{i}$, respectively.

*Definition* Average upper-limb activity is a construct that reflects of how long and how intensely an upper-limb is used in a given time period *D*.

High amounts of average upper-limb activity correspond to long duration, high intensity movements, while low activity corresponds to short duration, low intensity movements. Average upper-limb activity $A_{i}$ of the upper-limb *i* can be captured by the product of $U_{i}$ and $I_{i}$, which quantifies the co-variation of these two factors. We thus define $A_{i}$ as,

(6) $$\begin{array}{l} A_{i}\left(t\right)≜U_{i}\left(t\right)\cdot I_{i}\left(t\right)=\frac{1}{D}\int_{t-D}^{t}\mu_{i}\left(x\right)dx,\;t\in \left[D,T\right] \end{array}$$

where, $A_{i}\left(t\right)\in {\mathbb{R}}_{\geq 0}$ assumes non-negative values and is upper-bound by $I_{i}\left(t\right)$. A subject with high values for $A_{i}$ would be referred to as more active, than one with lower values of $A_{i}$. Visualization of how much an upper-limb is used during a measurement epoch, and its quantification through a single number using $A_{i}$ are discussed in section 4.2.

### 3.7. Functional Workspace

We are also often interested in knowing the region of space around a person's body where an upper-limb is used to reach and manipulate the environment (Ploderer et al., 2016). The in-clinic assessment of active range of motion of an upper-limb only tells us about the space that can be reached by a subject. It does not necessarily convey information about the space a subject regularly moves to carry out daily activities. We refer to the latter as the functional workspace, defined as the following.

*Definition* Functional workspace of an upper-limb is a quantitative representation of space traversed by an upper-limb when carrying out functional activities during daily life.

In this definition space could be the endpoint (Euclidean) space of the hand represented in an egocentric frame of reference, or the joint space upper-limb composed of the various joints of the limb.

Let $W_{i}\left(t\right)$ represent the functional workspace of upper-limb *i* computed at the time instant *t* from a segment of *M*_*i*_ from the last *D*sec, i.e., from *t* − *D* sec to *t* sec. In order to have a general and informative representation of the workspace, we define $W_{i}$ to be a probability density function over the space of interest, where the density at a given spatial location is proportional to the relative amount of time spent in a small volume of space around the spatial location during the last *D* sec of performing functional movements or postures.

(7) $$\begin{array}{l} W_{i}\left(t;D\right)=f_{W}\left(M_{i},u_{i};t,D\right) \end{array}$$

where, $f_{W}\left(M_{i},u_{i};t,D\right)$ is the function that computes a probability density function from the segment of the measurement *M*_*i*_ and upper-limb use *u*_*i*_ signals between times *t* − *D* and *t*. Such a probability density function would allow useful visualization of the functional workspace as a heatmap representing regions of space that a subject is comfortable traversing during their daily activities. Such heatmaps have been found to be useful by clinicians to understand the nature of use of the upper-limb (Ploderer et al., 2016). It should be noted that one might also be interested in the workspace of the limb when performing non-functional movements—non-functional workspace, which can be obtained from $f_{W}\left(M_{i},1-u_{i};t,D\right)$.

Additionally, the use of probability density functions for $W_{i}$ also enables one to compute various summary measures about the nature of the workspace, such as volume of the functional workspace, preferred spatial locations during day-to-day activities, symmetry of the functional workspace between the two limbs, etc., which help to characterize different aspects of the functional workspace.

### 3.8. Task

The constructs discussed so far—*u*_*i*_, μ_*i*_, $U_{i}$, $I_{i}$, $A_{i}$, and $W_{i}$—are task-agnostic constructs that only depend on whether or not a meaningful movement or posture is performed, irrespective of its type (e.g., reaching, manipulation, drawing). To elucidate the nature of upper-limb use, task-specific measures are required, i.e., measures that can classify the types of tasks being performed, how well these tasks are performed, etc. This information could be used to target therapy to accomplish specific rehabilitation goals. To carry out task-specific analysis, one must first define a set of tasks of interest that can be identified from the measurement *M*_*i*_.

*Definition* Task is any upper-limb movement or postural pattern of interest.

Let the set 𝕋 ≜ {0, 1, 2, …*p*} ⊂ ℕ be a set of natural numbers representing the *p* distinct tasks of interest; the numbers from 1 to *p* correspond to the *p* tasks, and 0 represents all tasks other than these *p* tasks of interest. Let τ_*i*_(*t*) ∈ 𝕋([0, *T*]) represent the task performed by the upper-limb *i* at time *t*.

(8) $$\tau_{i}≜f_{\tau }\left(M_{i}\right),\;\tau_{i}\left(t\right)=\{\begin{array}{ll} n, & \text{Task }n\text{ is being performed by UL }\\ \; & i\text{ at time }t.\\ 0, & \text{None of the n tasks of are being}\\ \; & \text{performed by UL}i\text{ at time }t. \end{array}$$

The function *f*_τ_ is a measure that maps the measurement signal *M*_*i*_ to τ_*i*_, i.e., *f*_τ_:𝕄([0, *T*]) ↦ 𝕋([0, *T*]). We assume that, in general, the *p* tasks of interest are functional in nature, which implies τ_*i*_(*t*) can take on a non-zero value only if *u*_*i*_(*t*) = 1. Similar to upper-limb use, the detection of tasks from the measurement data will also be probabilistic in nature due to the natural intra- and inter-subject movement variability (Bulling et al., 2014). Human activity recognition using various sensing modalities is a current area of research in the machine learning and artificial intelligence community (Bulling et al., 2014; Nweke et al., 2018). Wearable sensors such as IMUs and vision systems are two commonly employed sensing modalities for recognizing different activities or tasks. The choice of the algorithm *f*_τ_ will depend on several interrelated factors, all of which have a bearing on its overall performance in detecting tasks:

- the exact nature of the measurements 𝕄, determined by the types and number of sensors used for measuring movement behavior. A diverse set of sensing modalities and higher numbers of sensors is likely to result in better detection performance. For wearable sensors, there is evidence that indicates that sensors on multiple segments of the arm can result in better performance than a single sensor just on the hand (Bulling et al., 2014).
- the sets of tasks 𝕋 that one is interested in detecting. The choice of the specific tasks of interest is application specific, and must ensure they can be detected using the available sensor data. Ideally, one must avoid complex tasks which are composed of a set “sub-tasks” (e.g., cooking) and require contextual information about the user and the environment that is often not available (Van Meulen et al., 2016). Furthermore, the chosen tasks in 𝕋 must have distinct kinematic patterns that can be robustly distinguished with the available measurements.
- the size of annotated ground-truth data available for training a chosen algorithm will determine the nature of algorithm that can be used. This factor will also have direct implications for the generalizability of the algorithm's performance to unseen data, and thus its performance. The size of the training dataset required for any algorithm will depend on the nature of the algorithm, the number of tasks, and the intra- and inter-class variability in the data for the problem of interest. For example, machine learning algorithms perform better in detecting movement behavior in healthy subjects compared to patients (Lum et al., 2020), arguably due to an increased inter-subject (and may be also intra-subject) variability in patient populations.
- the availability of computational resources will also influence the types of algorithms that can be trained and used for detecting tasks. Real-time algorithms running on wearable devices will have more constraints (Laput and Harrison, 2019) than algorithms that work on the data offline on a PC.

A range of different algorithms have been explored for human activity recognition, such as Hidden Markov Models, Decision Trees, Supper Vector Machines, k-Nearest Neighbors, etc. (Bulling et al., 2014). More recently there has been an increased use of deep learning networks for activity recognition (Nweke et al., 2018), which have shown very promising results even with a single wrist-worn smartwatch measuring accelerations of the wrist (Laput and Harrison, 2019).

### 3.9. Movement Quality (MQ)

*Definition* Movement quality (MQ) is construct that reflects the quality of the underlying sensorimotor control.

MQ is a high level construct indicative of the motor ability of a user, and is composed of other constructs such as movement smoothness (Balasubramanian et al., 2015), coordination (Levin, 1996; Cirstea et al., 2003), tremor (Mansur et al., 2007), etc. Movement quality includes both tasks-specific and task-agnostic constructs, which differ primarily in terms of their computational procedure, and their interpretation. Task-agnostic measures of movement quality (e.g., amount of tremor) can be computed from the *M*_*i*_ without worrying about the underlying tasks being performed. For instance, the amount of tremor in a frequency band of interest could be computed over short segments of movement data from the entire measurement epoch. The numbers, thus obtained, indicate the change in the amount of tremor as function of time. Although there can be several reasons (e.g., the task currently performed) that influence the amount of tremor experienced by a subject, these reasons do not influence what the numbers mean at a given time instant. On the other hand, task-specific measures (e.g., smoothness, coordination, etc.) must be computed only from complete data segments corresponding to a particular occurrence of a specific task. The appropriate interpretation of such task-specific movement quality measures requires necessary contextual information, which must include at least the task being performed (Balasubramanian et al., 2015). For example, an equally smooth reaching movement and a drawing movement will result in two different values computed from the same smoothness measure, because the spatio-temporal constraints of the two tasks are different (Balasubramanian et al., 2015). Thus, for such task-specific constructs the numbers alone are not sufficient to appropriately interpret quality of the observed movement. The context in which the different tasks are performed are also crucial for meaningfully interpreting these numbers in this scenario (Ploderer et al., 2016).

Similar to the other constructs discussed above, issues such as the nature of the available sensor data, the use of appropriate measures for movement qualities, applicability of these measures with different types of sensor data etc. need to be considered. For instance, recent work on estimating movement smoothness with IMUs has shown that the SPARC measure cannot be used with acceleration data, even though the SPARC has been shown to be a good measure of movement smoothness when applied on movement velocity (Melendez-Calderon et al., 2021).

Given these difficulties, it is not surprising that there is currently little work on assessing movement quality of upper-limb functioning in daily life using sensors (Bulling et al., 2014). We note that it might also be of interest to evaluate the quality of postures (e.g., the amount of scapular elevation used by subject to hold an object against gravity), in which case movement quality can be generalized to mean movement or posture quality. Furthermore, common compensatory strategies, such as the use of the trunk to compensate for shoulder and elbow deficits (Levin et al., 2002, 2009), would also fall within the purview of movement quality. Such compensatory strategies can be seen as some form of task-specific abnormal coordination between the different joints of the trunk and upper-limb.

## 4. Visualization and Quantification of Upper-Limb Functioning

Measuring upper-limb movements during daily-life can result in vast amounts of data, which needs to be summarized through appropriate quantitative and graphical means. A well-designed graphical summary can provide quick and clear insights into data, and allow users to answer specific questions about upper-limb behavior. In this section, we present three graphical approaches for summarizing answers to Q1 and Q2 discussed in the introduction: (a) temporal profile of upper-limb functioning for depicting the variation of upper-limb use over time; (b) summary of upper-limb activity; and (c) relative use of the two upper-limbs. Each graphical approach mentioned above is explained using data obtained from a previous study by David et al. (2020). The measurements were obtained from IMUs donned on each wrist, i.e., $M_{i}\left(t\right)={\left[\begin{array}{cc} {\text{a}}_{i}{\left(t\right)}^{⊤} & \omega_{i}{\left(t\right)}^{⊤} \end{array}\right]}^{⊤}\in {\mathbb{R}}^{6}=𝕄$ and consists of the linear acceleration **a**_*i*_(*t*) and angular velocity ω_*i*_(*t*) measured by the triaxial accelerometer and gyroscope, respectively, at time *t* from the upper-limb *i*. Upper-limb use was estimated using the GM score algorithm (Leuenberger et al., 2017), and instantaneous intensity of upper-limb use was chosen to be the activity counts (Brønd et al., 2017) derived from the accelerometer data. Average upper-limb use and intensity were computed using *D* = 60*s*. Note that the visualizations discussed below are not restricted to one particular sensing modality but can be appropriately adapted for different measurements as discussed in section 5.1.

### 4.1. Temporal Profile of Upper-Limb Functioning

**Figure 3:**
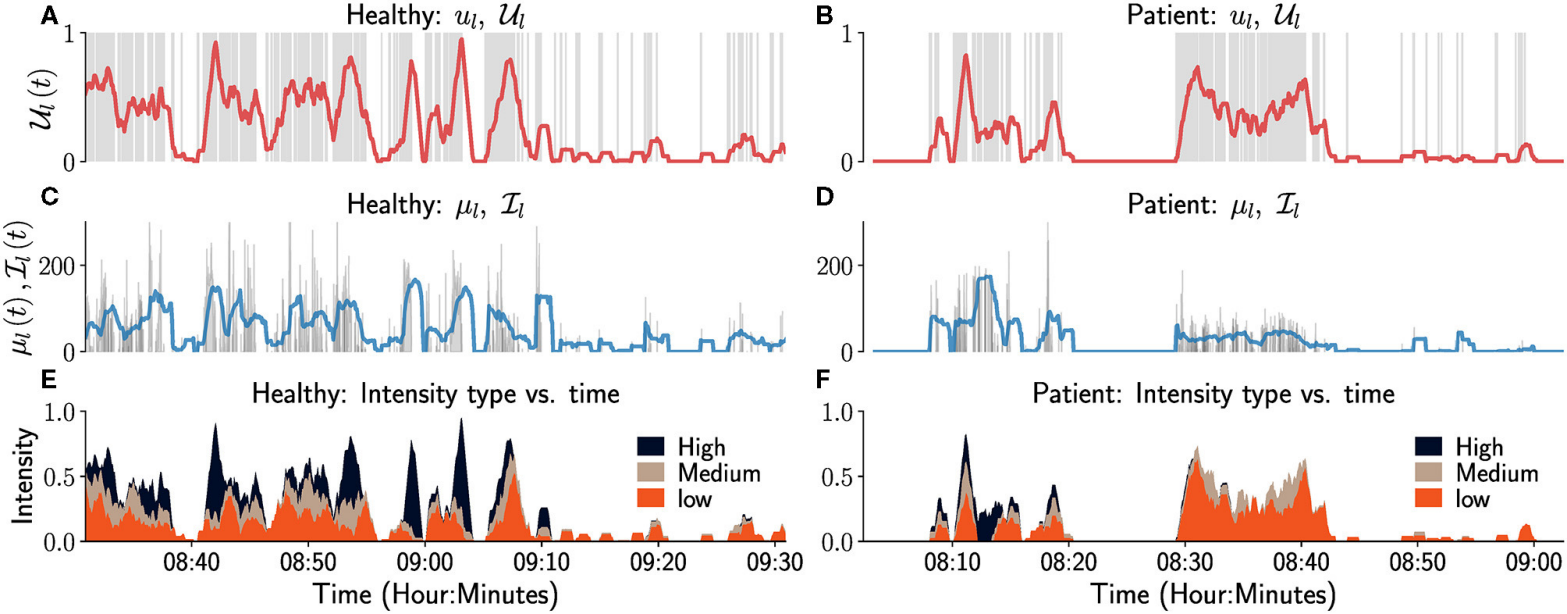
Temporal visualization of constructs related to left upper-limb use and intensity. The left and right columns correspond to data from a healthy participant and a patient, respectively. **(A,B)** depicts the left upper-limb use signal *u*_*l*_ as a gray-colored event plot, where the vertical gray line at time t indicates *u*_*l*_ *(t)* = 1. And the light red colored graph shows the corresponding average upper limb use $U_{l}$. **(C,D)** depicts the instantaneous intensity of use μ_*l*_ (gray) and the average intensity of use $I_{i}$ (light blue) for the left upper-limb. **(E,F)** depicts the proportion of time the intensity of use was low (orange), medium (brown), or high (black) in the last 60 s. Although not shown in these figure, it would also be useful to indicate in such plots periods of time where there is no data available, i.e., periods where a wearable sensor has been removed and is not recording movement data from a subject.

The plots of *u*_*i*_, μ_*i*_, $U_{i}$, $I_{i}$ over the course of the measurement epoch, allows the user to see variations in these constructs over the course of a day or days. Sample plots of *u*_*l*_, $U_{l}$, μ_*l*_, $I_{l}$ for a healthy (left column) and an impaired subject (right column) over a period of 90 min are shown in Figure 3. The left upper-limb use *u*_*l*_ is visualized as an event plot in Figures 3A,B, where the presence of a gray vertical line at time *t* means *u*_*l*_(*t*) = 1, else it is 0. The average upper-limb use or duration $U_{l}$ is displayed in a red trace in Figures 3A,B. Figures 3C,D display the corresponding μ_*l*_ and $I_{l}$ for this period in gray and blue traces, respectively. We note that μ_*l*_(*t*) = 0 in these plots correspond to either the upper-limb not being used or a functional posture, since both the GM score algorithm and the activity counts are insensitive to postures. Similarly, $I_{l}\left(t\right)=0$ when the upper-limb was not used or used in a posture in the last *D* seconds, i.e., $U_{l}\left(t\right)=0$.

$I_{l}\left(t\right)$ only provides a summary of the intensity of left upper-limb use in a temporal segment by computing the average intensity. A more detailed depiction of movement intensity can be provided by displaying the relative proportions of time the movement intensity is low, medium, or high, in an observation window; the definitions of the three intensity levels are provided in the figure's caption for this particular case. The plots in Figure 3 can aid clinical evaluation. It indicates that the overall amount and intensity of use for the patient (right column) is lower than that of the healthy subject. The patient also has little or no high intensity movements compared to the healthy participant (Figures 3E,F).

### 4.2. Visualization of Upper-Limb Activity

A visual summary of the amount of upper-limb use during a measurement epoch can be provided through a scatter plot of $U_{i}\left(t\right)$ vs. $I_{i}\left(t\right)$, ∀*t*, such that $U_{i}\left(t\right)\neq 0$^2^. This plot will be referred to as the Use vs. Intensity plot, UI plot, which provides a simple visual summary of the overall upper-limb activity. With no loss of generality, we have chosen $I_{i}$ and $U_{i}$ to be the *x* and *y* axes of the UI plot, respectively, which has the following properties:

- All points of this scatter plot belong to the set *P* = {(*x, y*)|0 ≥ *x*, 0 < *y* ≤ 1}. This a strip of height 1 extending along the positive *x* axis.
- By definition, the *x* axis is not part of the plot since only data points where $U_{i}\neq 0$ are considered.
- Depending on the measurement signal and the choice of measure *f*_μ_, the set of all points {(0, *y*)|0 < *y* ≤ 1} will correspond to upper-limb postures; this will not be true when $I_{i}\left(t\right)\neq 0$ for meaningful postures.
- Scatter points with large values for *x* and low values for *y* correspond to short duration high intensity movements, e.g., swatting a fly. Whereas, points with values of *y* close to 1 and low values for *x* correspond to prolonged low intensity movements, e.g., writing, typing.

**Figure 4:**
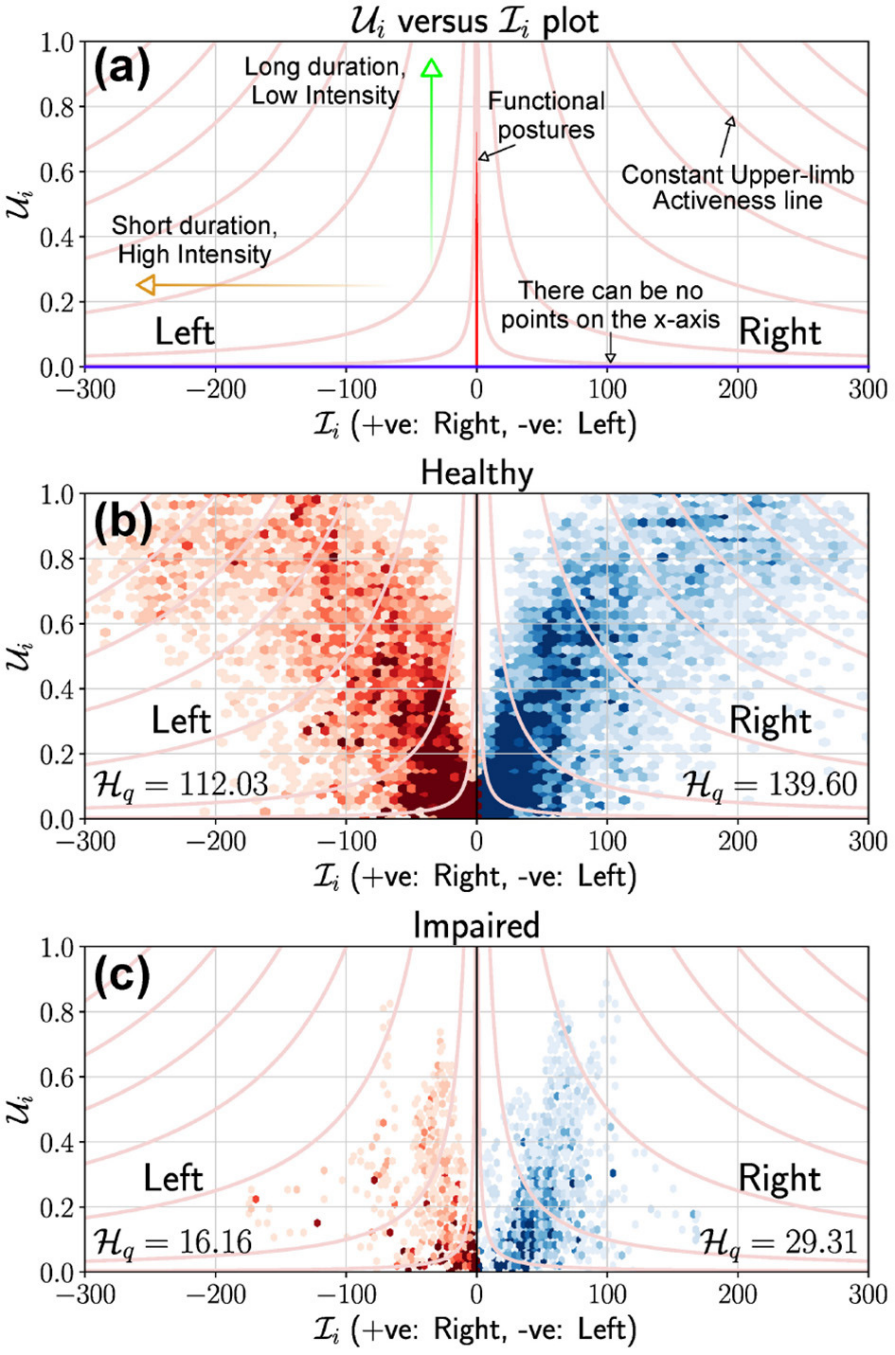
Use vs. Intensity (UI) plot to depict the overall amount of use of the upper-limbs. **(a)** This plot provides the details of a typical UI plot and highlights some critical elements to help interpretation. The *x* axis cannot be part of the plot, and light red colored curves are the constant upper-limb activity lines. If *f*_μ_ is the magnitude of acceleration as is the case in **(b,c)**, then the *y* axis represents meaningful/functional postures where the intensity can be zero. **(b)** UI plot for a healthy participant using data collected from a single day. The 1st and 2nd quadrants of the scatter plot depicts the right (blue) and left (red) upper-limbs, respectively. **(c)** UI plot for a stroke participant using data collected from a single day. It is clear that the stroke participant has a low level of activity compared to the healthy participant, which is also reflected in their corresponding $H_{q}$ scores.

Data from both upper-limbs can be visualized in a single plot by plotting them in the first and second quadrants as shown in Figure 4. Here, the right and left upper-limbs are depicted in the first and second quadrants, respectively; note that the data in the second quadrant are plotted by negating the value of $I_{i}$. The light red colored lines in these plots correspond to constant average upper-limb activity lines, i.e., $A_{i}=U_{i}\cdot I_{i}=c$, where *c* is a constant.

Figures 4b,c display the UI scatter plots for a healthy and stroke participant, respectively, using data collected from a single day (6–8 h) of recording (David et al., 2020). For the healthy subject, most points are of short to medium duration $\left(U_{i}<0.5\right)$ and low intensity $\left(I_{i}<50\right)$ in Figure 4b, with some long duration, high intensity movements performed with both limbs. In comparison, most movements of the stroke participant were of relatively shorter duration $\left(U_{i}<0.2\right)$, with low to medium intensity movements $\left(I_{i}<100\right)$; high intensity movements $I_{i}>100$ were rare. This observation is also evidenced by reduced number of constant $A_{i}$ lines that cut through the scatter plot in Figure 4c compared to that of the healthy subject.

### 4.3. Quantification of Overall Upper-Limb Activity

The distribution of points in an UI plot can be thought of as a sample obtained from a bi-variate probability density function of U and I, $p_{I,U}\left(x,y\right)$^3^. The univariate probability densities of $U_{i}$, $I_{i}$, and $A_{i}$ can be obtained from $p_{I,U}$ as the following,

(9) $$\begin{array}{l} p_{I}\left(x\right)=\int_{0}^{1}p_{I,U}\left(x,y\right)dy;\\ p_{U}\left(y\right)=\int_{0}^{\infty }p_{I,U}\left(x,y\right)dx;\\ p_{A}\left(z\right)=\int_{0}^{1}p_{I,U}\left(\frac{z}{y},y\right)\sqrt{1+\frac{z^{2}}{y^{4}}}dy \end{array}$$

We define a quantitative measure of how much an upper-limb is used, $H_{q}$, as the *q*^*th*^ percentile of A, which can be computed from its probability density function $p_{A}$,

(10) $$\begin{array}{l} H_{q}≜q_{A},\;\text{s.t.}\int_{0}^{q_{A}}p_{A}\left(z\right)dz=q \end{array}$$

where, the subscript in *q* in $H_{q}$ indicates that the measure is computed using the *q*^*th*^ percentile.

**Figure 5:**
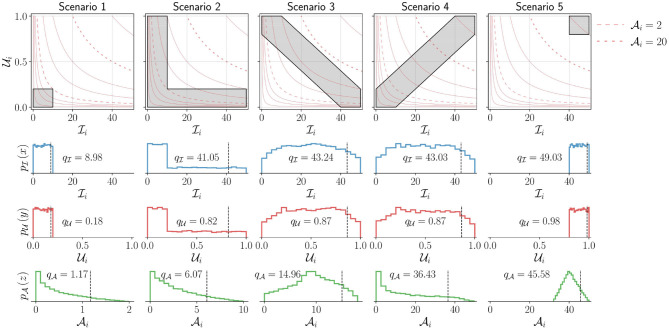
Demonstration of the measure $H_{q}$ for five different simulated scenarios corresponding to different levels of upper-limb activity. The top row shows the UI plot for the different scenarios. The shaded areas (gray) indicate different simulated scenarios from which points are sampled with uniform density. Two of constant activity lines (light red) in each plot are shown as dashed lines corresponding to $A_{i}=2$ and $A_{i}=20$. The bottom three rows depict the marginal probability density functions for $I_{i}$ (second row), $U_{i}$ (third row), and $A_{i}$ (bottom row) for these different scenarios. The black vertical dashed line indicates the *q*^*th*^ percentile (here, *q* = 90) for these different scenarios with the corresponding value written on the individual plots. Note that to enable the proper depiction of the density functions for the different scenarios, the scale for the *x* axis for the bottom row is adjusted.

**Properties of** $H_{\text{q}}$. We demonstrate through a set of simulated scenarios (Figure 5) that the measure $H_{q}$ agrees with our intuition. Consider the scenarios depicted in Figure 5, which shows five UI plots, in the top row, with different distribution of points. In each of these plots, points are assumed to be uniformly distributed in the gray regions shown; the light red colored curves are the $A_{i}=c$ lines, where *c* is a constant. The rows of plots below the UI plots show the univariate probability density functions $p_{I}$, $p_{U}$, and $p_{A}$ estimated from the data points sampled from the corresponding distributions $p_{I,U}$ shown in the UI plots in the top row, along with the corresponding *q*^*th*^ percentile values of the sample data (*q* was set to 90).

The following observations can be made about the five scenarios depicted in Figure 5, which are reflected in the measure $H_{q}$:

- Scenario-1 has the lowest upper-limb activity $\left(H_{q}=1.18\right)$ among all scenarios, as all movements are of short duration and low intensity. $U_{i}\in \left[0,0.2\right]$, $I_{i}\in \left[0,10\right]$, and $A_{i}\in \left[0,2\right]$.
- Scenario-5 has the highest upper-limb activity $\left(H_{q}=45.64\right)$ as all movements are of long duration and high intensity. $U_{i}\in \left[0.8,1\right]$, $I_{i}\in \left[40,50\right]$, and $A_{i}\in \left[32,50\right]$.
- Scenarios 2, 3, and 4 have the same range of values for $U_{i}$ and $I_{i}$ $\left(U_{i}\in \left[0,1\right],I_{i}\in \left[0,50\right]\right)$ with similar values for $q_{I}$ and $q_{U}$.
- Scenario-2 has higher activity $H_{q}=6.10$ than scenario-1 as it contains movements of larger duration or higher intensity in addition to movements similar to scenario-1. This results in larger values for $A_{i}\in \left[0,10\right]$ compared to scenario-1.
- Scenario-3 has higher activity $H_{q}=14.78$ than scenario-2 as it has longer duration and higher intensity movements than scenario-2, resulting in even larger range of values for $A_{i}\in \left[0,18\right]$ than scenario-2.
- Scenario-4 has movements with longer duration and higher intensity than scenarios 2 and 3, resulting in a large interval for the possible values of $A_{i}\in \left[0,50\right]$ compared to scenarios 2 and 3. Thus, resulting in a much higher level of activity, $H_{q}=36.82$.
- The difference in upper-limb activity between scenario-4 and scenario-5 is smaller than that of scenario-4 and scenario-3. Scenario-4 has more long duration and high intensity movements than scenario-3, but has more shorter duration and lower intensity movements than scenario-5. Scenario-5 only has longer duration and higher intensity movements.

### 4.4. Visualization of Relative Use of the Upper-Limbs

Visualizing the relative use of the upper-limbs has been explored through 2D scatter plots or heat-maps of different variables related to the use of the upper-limbs (Bailey et al., 2014; David et al., 2020). Relative upper-limb use can be visualized and quantified using measures of average upper-limb use $\left(U_{r},U_{l}\right)$, average upper-limb intensity $\left(I_{r},I_{l}\right)$ or average upper-limb activity $\left(A_{r},A_{l}\right)$; here, we use average upper-limb intensity for demonstration purposes. We only consider data points where at least one of the two upper-limbs was used, i.e., $I_{r}\left(t\right)+I_{l}\left(t\right)>0$^4^; it is meaningless to talk about relative use when neither upper-limb is used.

In general, relative use of the upper-limbs can be visualized by plotting two functions $g\left(I_{r},I_{l}\right)$ and $h\left(I_{r},I_{l}\right)$ of the subject's data along the *x* and *y* axis, respectively. These two function *g*(·) and *h*(·) will determine the nature of distribution of data points in this “*gh*” scatter plot and its fundamental properties. A qualitative understanding of these properties can be obtained from the following four family of curves *L*_1_ to *L*_4_ in the *gh* plot:

(11) $$\begin{array}{l} \begin{array}{c} \text{Average intensity of the left upper-limb is constant}\\ -L_{1}:I_{l}\left(t\right)=c\\ \text{Average intensity of the right upper-limb is constant}\\ -L_{2}:I_{r}\left(t\right)=c\\ \text{Ratio of average intensities of the two upper-limbs is constant}\\ -L_{3}:I_{l}\left(t\right)=c\cdot I_{r}\left(t\right)\\ \text{Product of average intensities of the two upper-limbs is constant}\\ -L_{4}:\;I_{l}\left(t\right)\cdot I_{r}\left(t\right)=c \end{array} \end{array}$$

where, *c* ∈ ℝ_≥0_. *L*_1_ and *L*_2_ are particularly useful in explaining the shape of the distribution of points in the different visualization plots, where the bounding curves of a scatter plot are generated from different *L*_1_ and *L*_2_ curves. We present the analysis of three visualization methods, the first one based on the work of Bailey et al. (2014), the second from the work of David et al. (2020), and the third one is a rotated version of second plot. Two additional visualization methods based on BMMR and LIRI are presented in Appendix A.

#### 4.4.1. Bilateral-Magnitude vs. Magnitude-Ratio (BMMR) Plot

This method proposed by Bailey et al. (2014, 2015) and Lang et al. (2017) used activity counting to plot a heatmap between the *magnitude ratio* (MR) and *bilateral magnitude* (BM),

(12) $$\begin{array}{l} \begin{array}{c} x\left(t\right)=g\left(I_{l},I_{r}\right)=\text{MR}\left(t\right)=\log\left(\frac{I_{r}\left(t\right)}{I_{l}\left(t\right)}\right);\;\text{MR}\left(t\right)\in \mathbb{R}\\ y\left(t\right)=h\left(I_{l},I_{r}\right)=\text{BM}\left(t\right)=I_{l}\left(t\right)+I_{r}\left(t\right);\;\text{BM}\left(t\right)\geq 0 \end{array} \end{array}$$

**Figure 6:**
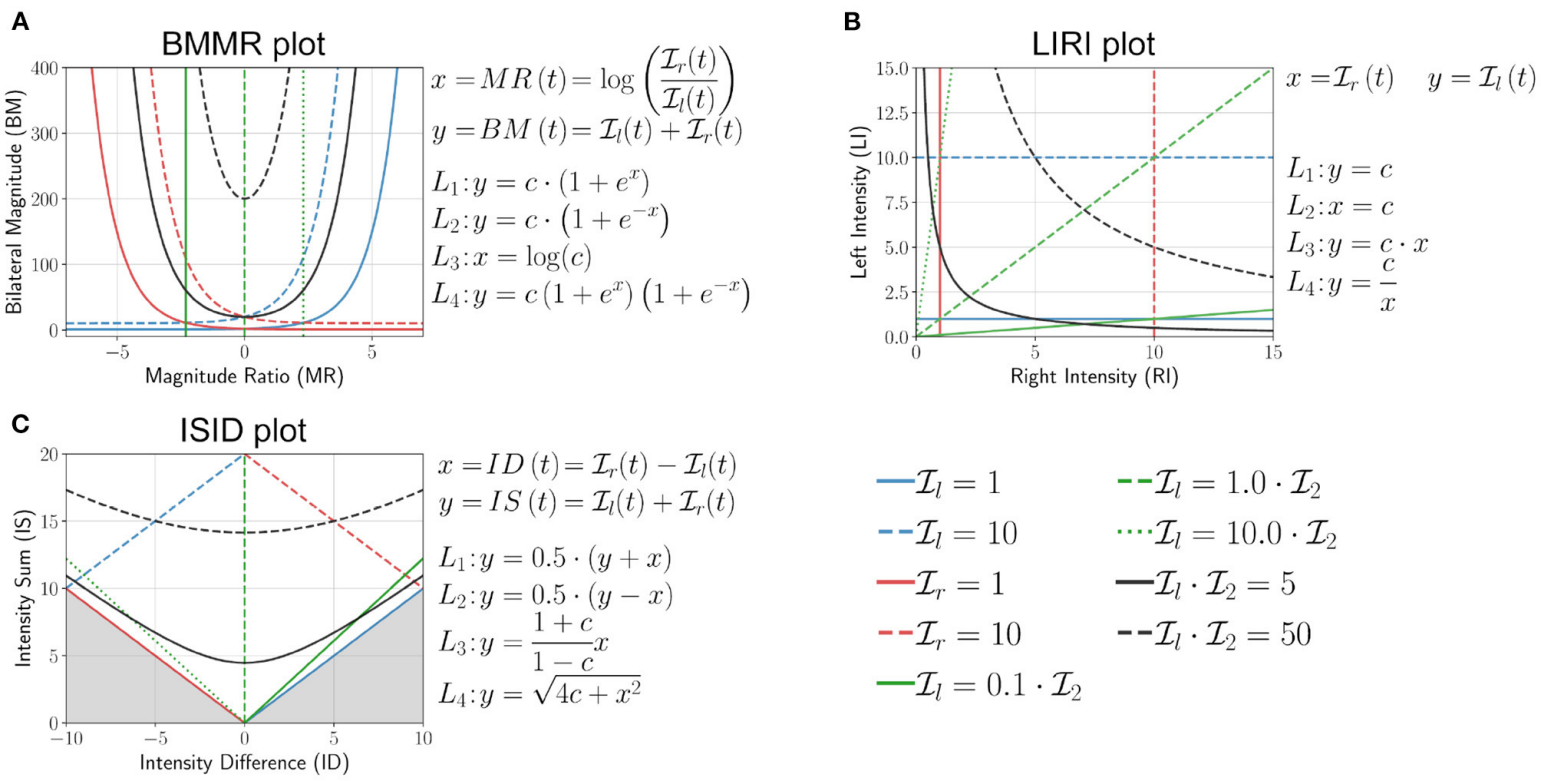
Analysis of **(A)** bilateral magnitude vs. magnitude ratio (BMMR) plot (Bailey et al., 2014), **(B)** left intensity vs. right intensity (LIRI) plot (David et al., 2020), and **(C)** intensity sum vs. intensity difference plot (ISID), by investigating the nature of the family of four curves *L*_1_ (blue), *L*_2_ (red), *L*_3_ (green), and *L*_4_ (black) introduced in Equation (11). The solid and dashed lines indicate different values of *c* for the same curve.

**Figure 7:**
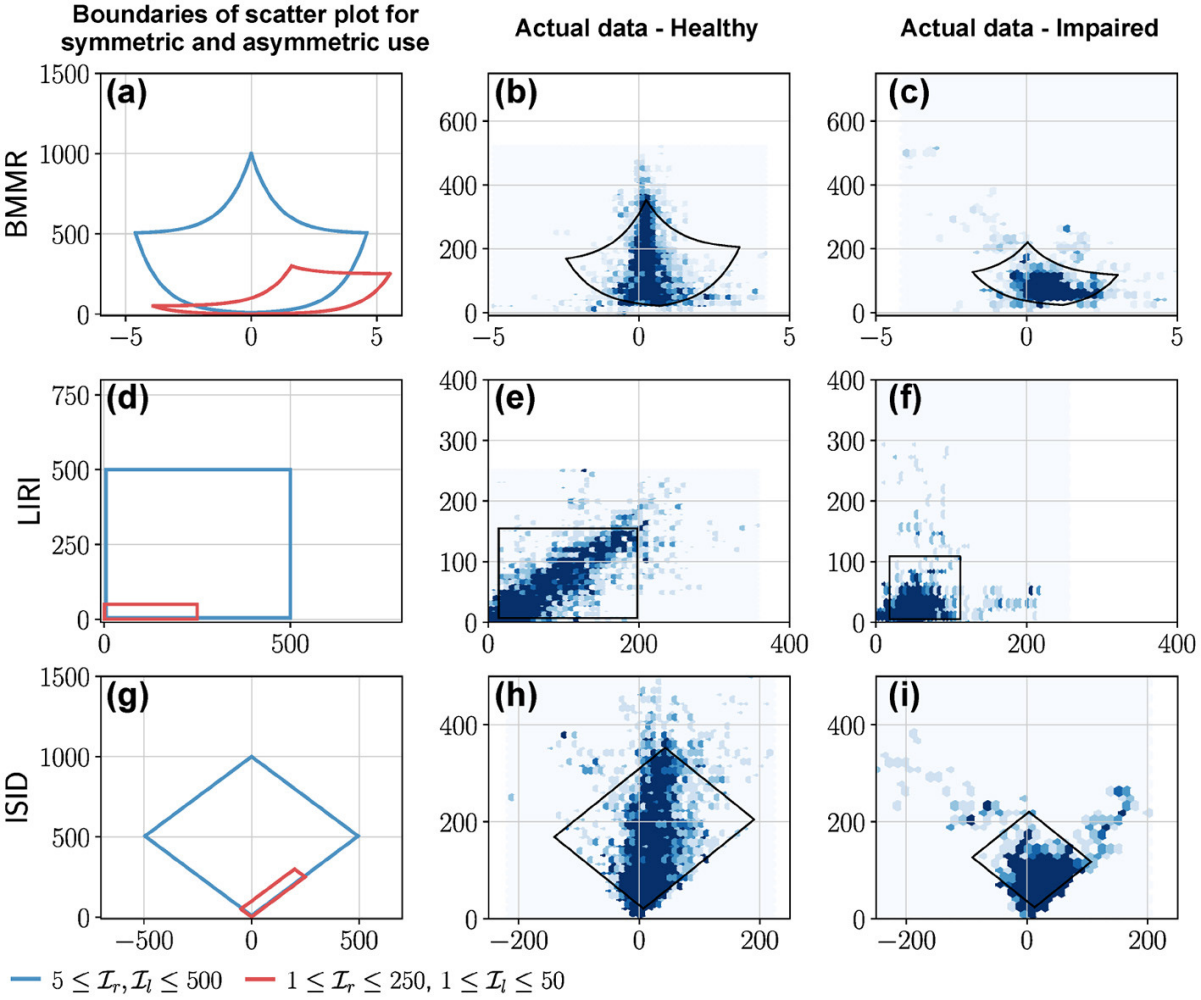
BMMR, LIRI, and ISID plots of actual data from a healthy participant and a patient. The first column shows examples of the boundary of scatter plots for **(a)** BMMR, **(d)** LIRI, and **(g)** ISID plots for symmetric and asymmetric upper-limb use. This closed curve corresponds to the *L*_1_ and *L*_2_ curves for different values of $I_{r}$ and $I_{l}$. **(a)** The symmetric leaf shape (blue) and the asymmetric (red) shape are typical shapes seen in the plots reported by Bailey et al. (2014). **(b,c)** Depict the BMMR scatter plots for a healthy participant and patient using data collected during a single day. **(e,f)** are the corresponding LIRI and **(h,i)** are the corresponding ISID, plots for the same subjects. The closed black curves shown in the plots for the healthy participant and patient correspond to the 2.5th and 97.5th percentiles for $I_{l}$ and $I_{r}$.

Bailey et al. bounded the value of MR to be within ±7, which we ignore in this discussion. The mathematical definitions of *L*_1_ to *L*_4_, and the plot of these curves for different values of *c* are shown in Figure 6A. The following are some of the essential properties of BMMR plot:

- The vertical line *x* = 0 corresponds to $I_{l}=I_{r}$, and divides the plot into two halves *x* > 0 and *x* < 0 corresponding to right and left dominated halves, respectively.
- Pure unilateral use $I_{l}=0$ or $I_{r}=0$ corresponds to *x* = ±∞, which was approximated to be *x* = ±7 by Bailey et al. (2014).
- Equal, unbiased use of the two upper-limbs results in a symmetric leaf-like distribution of points (blue curves in Figures 7a,b). The region enclosed by closed blue curve in Figure 7a corresponds to $5\leq I_{l},I_{r}\leq 500$. We note that the shape of the heatmaps for healthy subjects in Bailey et al. (2015) closely resembles this symmetric leaf shape.
- Biased use of the upper-limbs results in an asymmetric distribution of points, with more points located at a larger distance from the *x* axis on the side with increased use (red curve in Figures 7a,c). The region enclosed by closed red curve in Figure 7a corresponds to $1\leq I_{l}\leq 50$ and $1\leq I_{r}\leq 250$.

#### 4.4.2. Left Intensity vs. Right Intensity (LIRI) Plot

This simple approach was proposed by David et al. (2020) where the authors had used the average upper-limb use instead of intensity. Here, we use the average upper-limb intensity $I_{r}$ and $I_{l}$ (Figure 6B),

(13) $$\begin{array}{l} \begin{array}{c} x\left(t\right)=g\left(I_{l},I_{r}\right)=I_{r}\left(t\right);\;I_{r}\left(t\right)\geq 0\\ y\left(t\right)=h\left(I_{l},I_{r}\right)=I_{l}\left(t\right);\;I_{l}\left(t\right)\geq 0 \end{array} \end{array}$$

The following are some of the essential properties of the LIRI plot:

- The *y* = *x* corresponds to $I_{l}=I_{r}$ and divides the first quadrant into an upper and lower half about this diagonal line which correspond to relatively high left $I_{l}>I_{r}$ and right use $I_{l}<I_{r}$, respectively.
- Pure right and left unilateral use correspond to points long the *x* and *y* axes, respectively.
- Equal, unbiased use of the two upper-limbs in a square shaped region of distribution of points (blue curve in Figures 7g,h); the square is symmetric about the *y* = *x* line.
- Biased use of the upper-limbs results in rectangular distribution of points, with the longer side of the rectangular oriented along the axes corresponding to the upper-limb with increased use (red curve in Figures 7g,i).

#### 4.4.3. Intensity Sum vs. Intensity Difference (ISID) Plot

This plot is derived by rotating the LIRI plot by 45° counter-clockwise, which results in a plot of the sum vs. the difference between the average upper-limb intensities (Figure 6C).

(14) $$\begin{array}{l} \begin{array}{c} x\left(t\right)=g\left(I_{l},I_{r}\right)=\text{ID}\left(t\right)=I_{r}\left(t\right)-I_{l}\left(t\right);\;\text{ID}\left(t\right)\in \mathbb{R}\\ y\left(t\right)=h\left(I_{l},I_{r}\right)=\text{IS}\left(t\right)=I_{r}\left(t\right)+I_{l}\left(t\right);\;\text{IS}\left(t\right)\geq |ID\left(t\right)| \end{array} \end{array}$$

Because $I_{i}$ is non-negative, the points *y* < |*x*| are not part of the plot, which is shown by the shaded region in Figure 6C. The following are some of the essential properties of ISID plot:

- Like the BMMR plot, the *x* = 0 corresponds to $I_{l}=I_{r}$.
- Pure right and left unilateral use correspond to points long the *y* = *x* and *y* = −*x* lines, respectively.
- The shape of the distribution of points are the same as LIRI but are rotated by 45° counter-clockwise (Figures 7h,i).

### 4.5. Quantification of Relative Upper-Limb Use

A quantitative measure of relative upper-limb use should allow us to distinguish between different levels of relative use of the upper-limbs through a single number. Such a measure should map: (a) the spectrum of pure unimanual behavior to pure bimanual behavior to a compact interval on the real line, and (b) report low values for unimanual, and high values for bimanual behaviors.

We can conceive such a quantitative measure of relative upper-limb use through an approach similar to that of $H_{q}$. Consider the joint probability density $p_{I_{r},I_{l}}\left(r,l\right)$ of $I_{l}$ and $I_{r}$^5^. We can compute the marginal densities of $I_{r}$ and $I_{l}$, and the probability density of $I_{r}\cdot I_{l}$ from $p_{I_{r},I_{l}}\left(r,l\right)$ using the approach in Equation (9). We define a measure of relative upper-limb use $R_{q}$ as the following,

(15) $$\begin{array}{l} R_{q}\left(I_{r},I_{l}\right)≜\frac{q_{rl}}{\max\left(\overset{2}{\underset{r}{q}},q_{l}^{2}\right)} \end{array}$$

where, $R_{q}:{\mathbb{R}}_{\geq 0}\left[0,T\right]\times {\mathbb{R}}_{\geq 0}\left[0,T\right]\mapsto \left[0,1\right]$ maps two time signals $I_{r}$ and $I_{l}$ to the set [0, 1]. The subscript *q* in $R_{q}$ indicates that the measure is computed using the qth percentiles, and *q*_*r*_, *q*_*l*_ and *q*_*rl*_ are the qth percentiles of the probability density functions of $I_{r}$, $I_{l}$, and $I_{r}\cdot I_{l}$, respectively. It should be noted that *q*_*r*_ and *q*_*l*_ will never be simultaneously zero as we only include data points where $I_{l}\left(t\right)+I_{r}\left(t\right)>0$.

**Figure 8:**
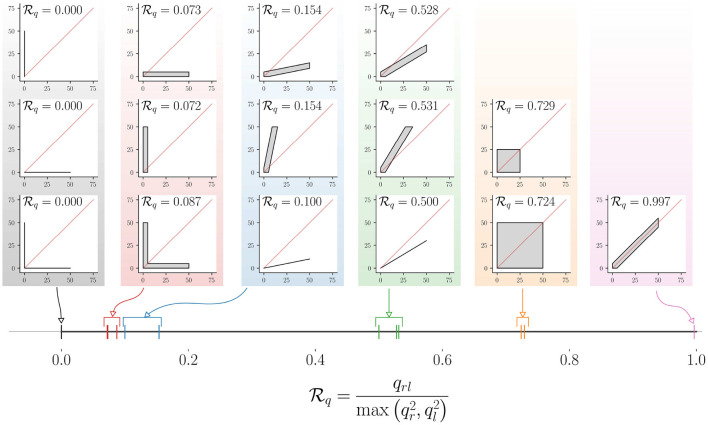
Demonstration of the mapping of different types of relative upper-limb use to $R_{q}$ (here, *q* = 90). The different types of relative upper-limb use are depicted as LIRI plots grouped together to into different levels of relative upper-limb use. The leftmost column of three LIRI plots correspond to pure unimanual use. The different groups of LIRI plots from left to right correspond to reduced bias in using one limb over the other. The corresponding $R_{q}$ value for these different scenarios are displayed in the individual LIRI plots, and their mapping to the continuous interval [0, 1] is shown in the bottom.

The mapping of different movement behaviors to the interval [0, 1] by this measure is shown in Figure 8, where the LIRI plot was chosen for depicting different types of unimanual and bimanual movement behaviors. The distribution of points in these LIRI plots are indicated by the gray regions, where we assume the points are distributed with uniform density; plots with just a black line depict scenarios where the points are distributed uniformly along the line. The red diagonal line in each of these LIRI plots is the *x* = *y* line. The value of $R_{q}$ for each of these plots is shown in the respective plots, and their location in the interval [0, 1] on the real-line is shown in the bottom of the figure (thick black line) with colored vertical lines. The $R_{q}$ measure has the following properties.

- **Pure unimanual use**. $R_{q}\left(I_{r},I_{l}\right)=0$ indicates pure unilateral use, such that $I_{r}\left(t\right)\cdot I_{l}\left(t\right)=0,\forall t$^6^.
- **Symmetric bimanual use**. $R_{q}\left(I_{r},I_{l}\right)=1$ indicates pure symmetric bimanual use, such that $I_{r}\left(t\right)=I_{l}\left(t\right),\forall t$.
- **Symmetry about the ***x*** = ***y*** line**. $R_{q}$ is symmetric about the *x* = *y* line, i.e., $R_{q}\left(I_{r},I_{l}\right)=R_{q}\left(I_{l},I_{r}\right)$. Two distribution of points that are mirror symmetric about the *x* = *y* line will have the same value for $R_{q}$. Thus, low values for $R_{q}$ only indicate biased use and do not provide any information about the direction of the bias. This implied that $R_{q}\left(I_{r},m\cdot I_{l}\right)=R_{q}\left(I_{r},\frac{1}{m}\cdot I_{l}\right)=m,0\leq m\leq 1$.
- $R_{q}$ **is independent of uniform scaling** $I_{r}$ **and** $I_{l}$, i.e. $R_{q}\left(I_{r},I_{l}\right)=R_{q}\left(c\cdot I_{r},c\cdot I_{l}\right),c>0$ is the value.

The measure $R_{q}$ only tells us if one limb is used over the other, and is silent about which of the two limbs is used more. This information can be obtained from the sign of the different between *q*_*r*_ and *q*_*l*_, which is +1 when the right limb is used more than the left, and −1 when it is vice versa. $R_{q}$ along with the sign of *q*_*r*_ − *q*_*l*_ will provide information amount of bias in using the upper-limbs, along with the preferred limb.

## 5. Discussion

The framework presented here is a step toward a rigorous foundation for the sensor-based assessment of upper-limb functioning by formalizing existing ideas/concepts. Lack of rigor is not an uncommon problem in movement science, which is reflected in the literature as ambiguous definitions of constructs, lack of clear specifications for measures, and absence of theoretical and experimental validation of measures proposed to quantify constructs of interest. Movement smoothness is a prime example of such a construct that was quantified using several measures with little or no knowledge about their properties (Balasubramanian et al., 2012, 2015). Given the increasing interest in assessment of upper-limb functioning using sensors, we strongly believe that the proposed framework can help guide future developments in this area. In this section, we highlight some important issues with sensor-based assessment of upper-limb functioning, and point out the limitations of the current work, and avenues for future work.

### 5.1. On the Importance of Measurements and Measures

Measurements and measures form the basis of any assessment procedure. Measurements contain “raw” information about an underlying behavior, and measures map measurements to numbers that quantify and summarize constructs of interest. Thus, the choice of measurements and measures determine the quality of information obtained from an assessment.

In the assessment of upper-limb functioning, several practical issues play a major role in the choice of sensing modality, such as the compactness, power efficiency, aesthetics, ease of donning and doffing of the sensors, privacy, etc. These constraints on a chosen sensing modality can limit the nature and fidelity of information about upper-limb functioning. For example, accelerometers have shown to perform a little better at detecting different tasks than gyroscopes alone (Bulling et al., 2014). Upper-limb use involving fine finger, wrist, and hand movements are unlikely to be captured by a single IMU worn on the forearm (Subash et al., 2020). Detecting tasks involving physical interactions with environment will require some form of vision technology (Tsai et al., 2020), and cannot be obtained purely from body segment kinematics. Similarly, the movement qualities that can be quantified also depend on the sensing modality, e.g., smoothness cannot be computed from pure accelerometer data, except under special circumstances (Melendez-Calderon et al., 2021).

Since these are early days in the field of assessment of upper-limb functioning, it would be unwise to make recommendations for the sensing modalities required for accurate assessment of upper-limb functioning in daily life. However, one can confidently speculate that a compact, body worn sensing system [e.g., wrist band (Bailey et al., 2014; David et al., 2020; Lum et al., 2020), sensorized clothing (Lorussi et al., 2016), etc.] with more than one sensing modality (Maceira-Elvira et al., 2019) [e.g., inertial sensor, pressure sensor, physiological sensing, vision, radar-on-a-chip for hand movement tracking (Malešević et al., 2019), magnetic ring finger tracking (Friedman et al., 2014) etc.] will become the standard for assessing upper-limb functioning.

### 5.2. On Task-Agnostic and Task-Specific Analysis of Upper-Limb Functioning

Upper-limb use *u*_*i*_ and instantaneous intensity of use μ_*i*_, their averages $\left(U_{i},I_{i},A_{i}\right)$, and the functional workspace $W_{i}$ together provide a measure of how much an upper-limb is used during a measurement epoch. These constructs are independent of the nature of the task being performed by the subjects, as they only demarcate functional behaviors from non-functional ones. The work presented in this manuscript focused only on task-agnostic analysis, given that these have been of primary interest in the recent literature. This is necessary information which only sheds light on the overall incorporation of the upper-limbs in daily life, without divulging the details of how the upper-limbs are used. Although, these task-agnostic construct provide some information about motor impairments, a more fine-grained task-specific analysis is required for identifying limitations in activity and participation.

Task-specific analysis requires the segmentation of measurements based on features of specific tasks of interest. Such analysis can allow the estimation of various impairment, activity, and participation level parameters to help build a comprehensive profile of the subject's disability. The details of the tasks performed during the measurement epoch provide information about limitations at the activity (e.g., time taken and range of motion while performing a task) and participation levels (e.g., limitations in carrying out household and work-related activities). The fidelity of such an analysis will depend on the nature of the available measurements, and algorithms that can accurately and robustly detect the task of interest. To our knowledge, there is currently no work on task-level analysis for assessing upper-limb movement functioning. This too is likely to change in the coming years with advances in human activity classification using sensors (Chen et al., 2020). Recent work by Schambra et al. on a taxonomy for upper-limb motion provides a nice framework for decomposing functional movements into different “functional primitives” (Schambra et al., 2019). They also demonstrated that most upper-limb functional activities carried out during therapy are captured by this taxonomy. One possible approach to leverage this work for task-specific analysis of upper-limb functioning is to develop algorithms to detect the five different functional primitives (reach, reposition, transport, stabilize, idle) defined in this taxonomy, and use these detected primitives to further identify higher level tasks/activities. This bottom up approach to detecting tasks/activities would also help quantify the “functional” composition of day-to-day movements in terms of functional primitives. Such a decomposition might be relevant for therapy planning, allowing therapist to focus therapy on primitives that might be limiting the patient's daily activity and participation.

### 5.3. On the Visualization of Upper-Limb Functioning

Ploderer et al. (2016) investigated the usefulness of different visualization methods for conveying information about upper-limb functioning in daily life. They found that temporal plots of the amount of upper-limb activity (similar to Figure 3) can be useful in understanding the use of the upper-limb from over several hours to weeks. The clinicians also emphasized the importance of a visualization method in providing a quick overview of the upper-limb functioning (Ploderer et al., 2016) over those provided by Use vs. Intensity (UI) and the relative upper-limb use plots in Figures 4, 7. Plots of functional workspace in the form of range of motion plots of joint angles and/or heatmaps of hand position in an egocentric frame were also found to be useful, which were not explored in the current study; data from a single wrist-worn IMU does not allow the extraction of hand position information or the arm joint angles. This again highlights the importance of the sensing modality in determining the information that can be obtained about upper-limb functioning.

The Use vs. Intensity plot, UI plot provides information about how much the upper-limbs are used during the measurement epoch, taking into account both the duration $U_{i}$ and intensity $I_{i}$ of movements. The nature of distribution of points in a UI plot depends on: (a) the nature of measurements *M*; (b) the function *f*_*u*_, *f*_μ_ used to quantify *u*_*i*_ and μ_*i*_; and (c) the window length *D* (Equations 4 and 5) used to compute $U_{i}$ and $I_{i}$. The extent to which the choice of these parameters affects interpretation were not investigated in this paper and requires further investigation.

Three approaches for visualizing relative use of the upper-limbs were analyzed in this paper. To promote the development and standardization of an appropriate visualization method, we make the following recommendations:

- **Avoiding complex transformations will make it easier to interpret graphs**. The LIRI plot is simpler than the BMMR plot, as $I_{l}$ and $I_{r}$ are visualized without any non-linear transformations. BMMR, MPMR, and BIUNI plots require complex transformations that hinder intuitive interpretation of these plots.
- **Symmetry about the ***x*** = 0 line might be easier to interpret**. Plots where the *x* = 0 line corresponds to $I_{l}=I_{r}$ divide the plot into two regions where the use of one upper-limb is higher than the other. These plots are easier to interpret. For instance, ISID plot, which is a rotated version of LIRI, is probably easier to interpret than LIRI.
- **Elucidating the properties of a visualization method**. Understanding a new visualization method can be made easier by depicting plots of special cases. For instance, the family of four curves *L*_1_ to *L*_4_ (Equation 11) were used to demonstrate some properties of the visualization approaches for relative use of the upper-limb. Thus, we recommend that researchers make use of such an approach when developing new visualization methods.

The visualization and quantification of relative use of the upper-limb were demonstrated using $\left(I_{r},I_{l}\right)$. Although the properties of the visualization and quantification using $\left(U_{r},U_{l}\right)$ or $\left(A_{r},A_{l}\right)$ are likely to be similar, there will be some differences. One must be cautious of these differences to ensure proper interpretation of the data. For example, unlike $I_{i}$ and $A_{i}$ the LIRI plot with $\left(U_{r},U_{l}\right)$ is restricted to the square 0 ≤ *x, y* ≤ 1.

### 5.4. On the Clinical Relevance of the Proposed Framework

Information about how an individual uses their upper limbs in every day activities is arguably a fundamental criterion of interest to a clinician. Our proposed framework aims at removing the ambiguity of what is meant by upper-limb functioning by defining key components that are necessary to depict “how” individuals behave in every day life in an objective manner. This information is conveyed by upper-limb use, intensity, and their averages, which relate to the overall duration and strenuousness of upper-limb use. The combination of these two constructs provide a good measure of how active an upper-limb is during daily life. Visualization of this information over the course of the day or across days was found to be useful by clinicians for monitoring upper-limb use in daily life (Ploderer et al., 2016). Asymmetry of upper-limb use can be evaluated from the level of activity of the two limbs which, when compared to normative data, can provide the measure of compensation employed by the patient by using the less affected limb. The ability to measure this asymmetry can help identify the underlying cause of this asymmetry, such as specific sensorimotor impairments, learned non-use (Taub et al., 2006), etc. The detailed characterization of upper-limb use by decomposing it into different tasks can provide ecologically relevant information about activity and participation. Furthermore, the time of occurrence, duration, and frequency of different tasks identified during daily life, and tracking these parameters over time can help evaluate changes in the ability and confidence in using the upper-limb either due to therapeutic interventions or spontaneous recovery. Finally, the quality of movement performed while carrying out different tasks can provide additional clues about the sensorimotor control ability and its relationship with hand preference and behavior.

The realization of the clinical utility of the different constructs in this framework and their incorporation in routine clinical use is at least a few years away, given the numerous technical and clinical hurdles that need to be overcome. To this end, we note some of the limitation of the current work and make suggestions for future research in the following subsection.

### 5.5. Limitations

This work is an initial attempt toward a framework for the systematic analysis and interpretation of upper-limb functioning using sensors. We hope that the ideas presented here form a base for future work in this area, and anticipate that these ideas will be further refined and improved in the coming years. To aid this process, we make explicit the limitations of the current work, which are as follows:

- The ideas presented in this work are theoretical in nature, and do not provide any specific algorithms or methods for quantifying the different constructs. Appropriate algorithms for realizing the measures *f*_*u*_, *f*_μ_, $f_{W}$, and *f*_τ_ are essential for practical implementation of a good assessment procedure, which will be an active area of research in the coming years.
- The different components of the proposed framework were chosen based on the authors' experience and understanding of the current clinical needs, and the trends in the neurorehabilitation literature. However, the clinical utility of these ideas (concepts, measure, and visualization methods) needs further validation.
- The work targets the evaluation of upper-limb functioning in hemiparesis. Thus, not all of the ideas presented here would be relevant for other conditions, such as those involving tremors, chorea, dystonia, etc. Application of this framework to other conditions, e.g., Parkinson's disease or orthopaedics, might require new concepts or revised definitions.
- Assessments of upper-limb functioning using sensors usually results in large amounts of data. The analysis methods that have been employed in the current literature and proposed in the current paper typically only extract a portion of information available in the measured data. Future work must focus on exploring data mining algorithms for identifying patterns of recurring behavior across time. Recent developments in computational ethology (von Ziegler et al., 2020) and automatic behavioral clustering (Berman et al., 2014) could be leveraged to identify such patterns. There is also currently little work on investigating patterns of upper-limb functioning within and across days, which might be useful in evaluating the participation of a patient in different day-to-day activities and their life roles.
- The work only addresses questions Q1 and Q2 presented in the introduction section, which deal with how much the upper-limbs are used in daily life and the bias in using one limb over the other. More detailed task-level analysis are likely to be of increasing interest in the future. Further, the work also did not explore measures for constructs such as “ability,” which may be of interest to a clinician; “ability” is likely to depend on amount of activity, types of tasks, and movement quality.

To address the aforementioned limitations and to advance the state-of-the-art in sensor-based assessment of upper-limb functioning, we make the following suggestions for future research:

- **Multi-modal sensing system**. Compact wrist/forearm-worn inertial sensors have been most popular solution to measuring upper-limb movements in recent times. Given the popularity this form-factor, exploring the use of additional sensing modalities such as radar-on-a-chip, EMG sensing, etc. for picking hand movements might be useful. Other sensing modalities such a textile-based sensing (Lorussi et al., 2016), egocentric camera (Tsai et al., 2020), and wrist-mounted cameras (Chen et al., 2018) might be able to provide more information about full body movements and object interactions.
- **Robust, accurate methods for detecting upper-limb use and tasks**. Recent work from Lum et al. (2020) and Subash et al. (2020) have carried out direct comparison of existing methods for detecting upper-limb use. This line of work, along with the development, validation and comparison of more sophisticated methods, leveraging the recent developments in machine learning, should be pursued to improve the accuracy and robustness of detecting upper-limb use and tasks of interest. One possible approach is to adapt ideas from human activity recognition literature to the specific needs of assessing upper-limb functioning. For instance, the taxonomy proposed by Schambra et al. (2019) could be used for building an algorithm that exploits the hierarchical structure of complex activities proposed in this taxonomy. Low-level algorithms can be devised for detecting the occurrence and duration of functional primitives. Information about the timing, duration, and amplitudes of these functional primitives could be used to detect the occurrence of more complex activities. The hierarchical analysis of sensor data might also be beneficial in identifying specific movement difficulties during daily life.
- **Open dataset of upper-limb behavior**. The development and validation of algorithms proposed in the previous points require data from the target population. The neurorehabilitation research community would immensely benefit from the availability of annotated open dataset consisting of relevant movement behaviors of interest from healthy and patient population with varying degrees of impairment. The development of such datasets and the sharing of data from various studies carried out in the community in this area can help drive the field forward in the coming years. The ImageNet (Deng et al., 2009) dataset played a crucial role in recent success of object recognition models in computer vision using machine learning algorithms. Similar efforts are already being made in the human activity recognition community (Laput and Harrison, 2019).
- **In-clinic and home-based clinical trials**. The clinical usefulness of the framework needs to be evaluated through both in-clinic and home-based studies. In-clinic studies for tracking upper-limb functioning of in-patients undergoing therapy would be relatively easier to carry out, and the availability of some information about the day-to-day routine of patients would allow validation of the assessment of upper-limb functioning using sensors. These can be followed by home-based studies to evaluate the usefulness of the proposed framework for assessing upper-limb functionally in the natural setting.

## 6. Conclusion

The paper presented a framework for sensor-based assessment upper-limb functioning, with focus on hemiparesis. The proposed framework provided formal definitions of constructs in upper-limb functioning, methods for their visualization, and two generic measures for quantifying the amount and the bias in using the two upper-limbs. Demonstration of some of these components were provided through preliminary data obtained from a previous study. We also pointed out the limitations of the current work which are likely to be addressed in the coming years. We firmly believe that the proposed framework can act as a scaffold for researchers in the field to build and test different ideas for assessment of upper-limb functioning. These future explorations will help identify issues with the framework, while adding, revising, and even completely replacing elements from the framework, which are not clinically and technically relevant. We hope this work is a useful step toward realizing an objective, accurate, and clinically relevant assessment tool to evaluate the true effect of neurorehabilitation in patients' daily life.

## Data Availability Statement

The data analyzed in this study was obtained from a previously published article (David et al., 2020). Requests to access these datasets should be directed to Sivakumar Balasubramanian, siva82kb@cmcvellore.ac.in.

## Author Contributions

AD and SB conceived the initial skeleton for the framework. The details of the framework were developed through discussions among AD, TS, SV, AM-C, and SB. AD and TS carried out the data collection and analysis presented in the paper. The initial manuscript was prepared by AD and SB. AD, TS, SV, AM-C, and SB reviewed, revised, and approved the final manuscript. All authors contributed to the article and approved the submitted version.

## Conflict of Interest

The authors declare that the research was conducted in the absence of any commercial or financial relationships that could be construed as a potential conflict of interest.

## Figures and Tables

**Figure A 1:**
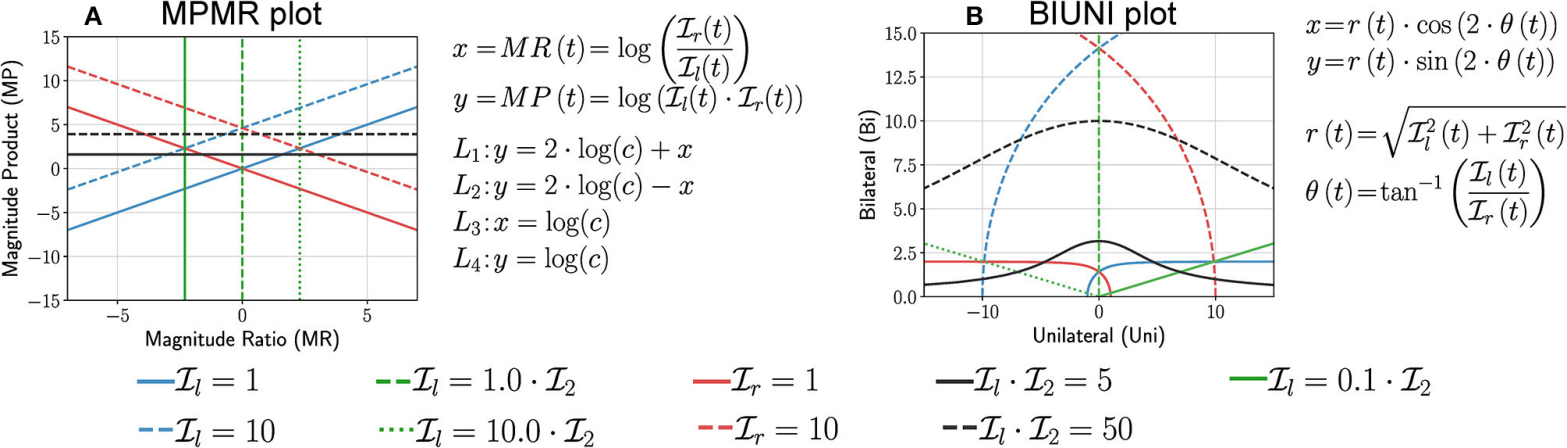
Analysis of MPMR and BIUNI plots by investigating the nature of the family of four curves *L*_1_ to *L*_4_ introduced in Equation (11). **(A,B)** Show the loci for different curves corresponding to *L*_1_ to *L*_4_ for the MPMR and BIUNI plots, respectively.

**Figure A 2:**
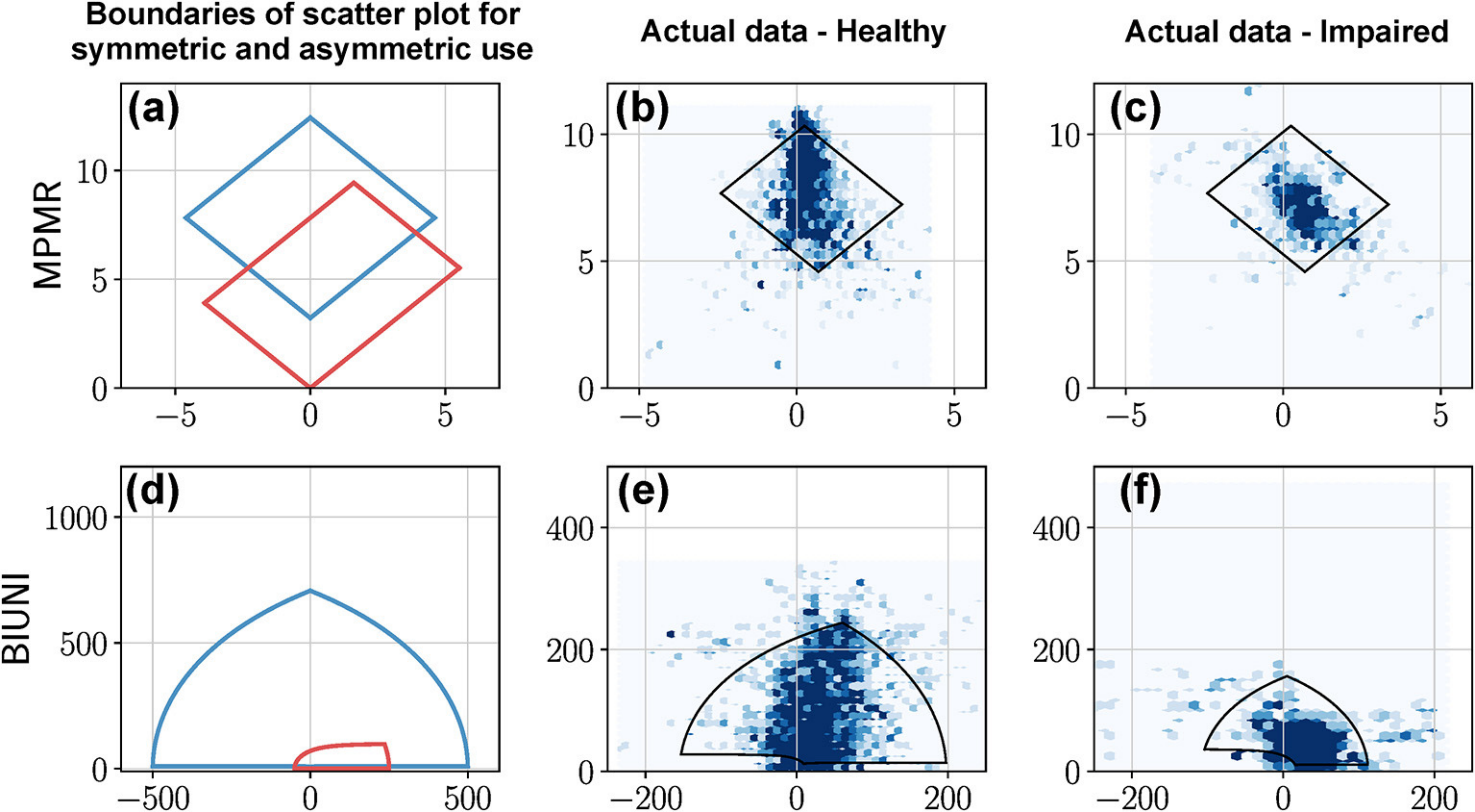
MPMR, and BIUNI plots of actual data from a healthy and impaired participant. The first column shows **(a,d)** examples of the boundary of the distribution of scatter plots for the MPMR and BIUNI plots for symmetric and asymmetric upper-limb use. This closed curve corresponds to the *L*_1_ and *L*_2_ curves for different values of $I_{r}$ and $I_{l}$. **(b,c)** Depict the MPMR scatter plots for a healthy and impaired participant using data collected during a single day. **(e,f)** Corresponding BIUNI plots for the same subjects. The closed black curves shown in the plots for the healthy and impaired participant correspond to the 2.5th and 97.5th percentiles for $I_{l}$ and $I_{r}$.

## Footnotes

^1^IMU—Inertial Measurement Unit—consists of an accelerometer and a gyroscope.

^2^This condition ensures that data segments where the upper-limb is at rest or is being used for non-functional movements are ignored.

^3^$p_{I,U}\left(x,y\right)$ is actually a conditional density function, since we only consider data where U≠0.

^4^As discussed earlier, $I_{i}$ can be zero during functional postures for some measurement signals and function *f*_μ_. Thus, under this scenario the condition $I_{l}\left(t\right)+I_{r}\left(t\right)=0$ will include data either when both upper-limbs are not used or when both are used for performing functional postures.

^5^This is again a conditional probability density function as we only consider data points where at least one of the upper-limbs is used, i.e., $I_{r}\left(t\right)+I_{l}\left(t\right)>0$.

^6^It should be noted that, depending on the measurements and *f*_μ_, pure unimanual does not always mean the other limb is not used, since $I_{i}$ can be zero when an upper-limb is used for functional postures.

## Appendix A

In this section we present analysis of two additional visualization methods by deriving expressions for the loci of *L*_1_ to *L*_4_, and demonstrate the nature of these visualization methods using data from a healthy and impaired participant (David et al., 2020) (similar to Figure 7).

### Magnitude-Product vs. Magnitude-Ratio (MPMR) Plot

Instead of using the sum of *I*_*l*_ and *I*_*r*_ along the *y* axis, like in BMMR, we can use the log of the product (MP) of the *I*_*l*_ and *I*_*r*_ (Figure A1A).

(16) $$\begin{array}{l} \begin{array}{c} x\left(t\right)=\text{MR}\left(t\right)=\log\left(\frac{I_{r}\left(t\right)}{I_{l}\left(t\right)}\right);\;\text{MR}\left(t\right)\in \mathbb{R}\\ y\left(t\right)=\text{MP}\left(t\right)=\log\left(I_{l}\left(t\right)\cdot I_{r}\left(t\right)\right);\;\text{MP}\left(t\right)\in \mathbb{R} \end{array} \end{array}$$

Some of the essential properties of the MPMR plot are:

- Like BMMR, the vertical line *x* = 0 divides the plot into right and left dominated halves (*x* > 0 for right and *x* < 0 for left).
- Pure unilateral use $I_{l}=0$ or $I_{r}=0$ corresponds to *x* = ±∞.
- The use of logarithmic transformation for the *x* and *y* axes leads to simpler looking curves for *L*_1_ and *L*_2_, and consequently simpler bounding regions as seen in Figure A2a.
- Equal, unbiased use of the upper-limbs results in a square-shaped region of distribution of points that is symmetric about the *x* = 0 line (blue curve in Figures A2a,b).
- Biased use of the upper-limbs results in a rotated rectangular region of points, with more of the rectangle located on one side of the vertical line (red curve in Figures A2a,c).

### Bimanual vs. Unimanual (BIUNI) Plot

BIUNI plot is obtained through a nonlinear transformation of the LIRI plot such that any point (*x, y*) in the LIRI plot with polar coordinates (*r*, θ) is mapped to a point (*r*, 2θ) (Figure A1B).

(17) $$\begin{array}{l} \begin{array}{c} x\left(t\right)=g\left(I_{l},I_{r}\right)=r\left(t\right)\cdot \cos\left(2\theta \left(t\right)\right);\;x\left(t\right)\in \mathbb{R}\\ y\left(t\right)=h\left(I_{l},I_{r}\right)=r\left(t\right)\cdot \sin\left(2\theta \left(t\right)\right);\;y\geq 0 \end{array} \end{array}$$

where, $r\left(t\right)=\sqrt{I_{r}^{2}\left(t\right)+I_{l}^{2}\left(t\right)}$ and $\theta \left(t\right)=\arctan2\left(I_{l}\left(t\right),I_{r}\left(t\right)\right)$.

Some of the essential properties of BIUNI plot are:

- Like the BMMR, MPMR, ISID plots, the *x* = 0 corresponds to $I_{l}=I_{r}$.
- Pure right and left unilateral use correspond to points long the positive and negative *x* axes, respectively.
- Equal, unbiased use of the upper-limbs results in a symmetric dome-shaped region of distribution of points (Figures A2d,e).
- Biased use of the upper-limbs distorts this shape resulting in more points distributed along the side of increased use (Figures A2d,f).

## References

[B1] André J.-M. Didier J.-P. Paysant J. (2004). Functional motor amnesia in stroke (1904) and “learned non-use phenomenon” (1966). *J. Rehabil. Med*. 36, 138–140. 10.1080/16501970410026107 15209457

[B2] Bailey R. R. Klaesner J. W. Lang C. E. (2014). An accelerometry-based methodology for assessment of real-world bilateral upper extremity activity. PLoS ONE 9:e103135. 10.1371/journal.pone.0103135 25068258 PMC4113366

[B3] Bailey R. R. Klaesner J. W. Lang C. E. (2015). Quantifying real-world upper-limb activity in nondisabled adults and adults with chronic stroke. Neurorehabil. Neural Repair 29, 969–978. 10.1177/1545968315583720 25896988 PMC4615281

[B4] Balasubramanian S. Melendez-Calderon A. Burdet E. (2012). A robust and sensitive metric for quantifying movement smoothness. IEEE Trans. Biomed. Eng. 59, 2126–2136. 10.1109/TBME.2011.2179545 22180502

[B5] Balasubramanian S. Melendez-Calderon A. Roby-Brami A. Burdet E. (2015). On the analysis of movement smoothness. J. NeuroEng. Rehabil. 12, 1–11. 10.1186/s12984-015-0090-9 26651329 PMC4674971

[B6] Barrett M. Snow J. C. Kirkland M. C. Kelly L. P. Gehue M. Downer M. B.. (2018). Excessive sedentary time during in-patient stroke rehabilitation. Top. Stroke Rehabil. 25, 366–374. 10.1080/10749357.2018.1458461 29609499

[B7] Berman G. J. Choi D. M. Bialek W. Shaevitz J. W. (2014). Mapping the stereotyped behaviour of freely moving fruit flies. J. R. Soc. Interface 11:20140672. 10.1098/rsif.2014.0672 25142523 PMC4233753

[B8] Blouin J.-S. Fitzpatrick R. C. (2010). Swing those arms: automatic movement controlled by the cerebral cortex. J. Physiol. 588(Pt 7):1029. 10.1113/jphysiol.2010.188649 20360024 PMC2852989

[B9] Bochniewicz E. M. Emmer G. McLeod A. Barth J. Dromerick A. W. Lum P. (2017). Measuring functional arm movement after stroke using a single wrist-worn sensor and machine learning. J. Stroke Cerebrovasc. Dis. 26, 2880–2887. 10.1016/j.jstrokecerebrovasdis.2017.07.004 28781056

[B10] Brønd J. Andersen L. Arvidsson D. (2017). Generating actigraph counts from raw acceleration recorded by an alternative monitor. Med. Sci. Sports Exerc. 49, 2351–2360. 10.1249/MSS.0000000000001344 28604558

[B11] Bulling A. Blanke U. Schiele B. (2014). A tutorial on human activity recognition using body-worn inertial sensors. ACM Comput. Surveys 46, 1–33. 10.1145/2499621

[B12] Chen F. Deng J. Pang Z. Baghaei Nejad M. Yang H. Yang G. (2018). Finger angle-based hand gesture recognition for smart infrastructure using wearable wrist-worn camera. Appl. Sci. 8:369. 10.3390/app8030369

[B13] Chen K. Zhang D. Yao L. Guo B. Yu Z. Liu Y. (2020). Deep learning for sensor-based human activity recognition: overview, challenges and opportunities. arXiv preprint arXiv:2001.07416.

[B14] Cirstea M. C. Mitnitski A. B. Feldman A. G. Levin M. F. (2003). Interjoint coordination dynamics during reaching in stroke. Exp. Brain Res. 151, 289–300. 10.1007/s00221-003-1438-0 12819841

[B15] David A. ReethaJanetSureka S. Gayathri S. Annamalai S. J. Samuelkamleshkumar S. Kuruvilla A.. (2020). Quantification of the relative arm-use in patients with hemiparesis using inertial measurement units. medRxiv [Preprint]. 10.1101/2020.06.09.20121996 PMC8273871 34290880

[B16] David A. Subash T. Varadhan S. Melendez-Calderon A. Balasubramanian S. (2021). A framework for sensor-based assessment of upper-limb functioning. bioRxiv [Preprint]. 10.1101/2021.02.10.430700 PMC8341809 34366809

[B17] de Lucena D. S. Stoller O. Rowe J. B. Chan V. Reinkensmeyer D. J. (2017). Wearable sensing for rehabilitation after stroke: bimanual jerk asymmetry encodes unique information about the variability of upper extremity recovery, in 2017 International Conference on Rehabilitation Robotics (ICORR) (London), 1603–1608. 10.1109/ICORR.2017.8009477 28814049

[B18] De Wit L. Putman K. Dejaeger E. Baert I. Berman P. Bogaerts K.. (2005). Use of time by stroke patients: a comparison of four European rehabilitation centers. Stroke 36, 1977–1983. 10.1161/01.STR.0000177871.59003.e3 16081860

[B19] Deng J. Dong W. Socher R. Li L.-J. Li K. Fei-Fei L. (2009). ImageNet: a large-scale hierarchical image database, in CVPR09 (Miami, FL). 10.1109/CVPR.2009.5206848

[B20] Friedman N. Rowe J. B. Reinkensmeyer D. J. Bachman M. (2014). The manumeter: a wearable device for monitoring daily use of the wrist and fingers. IEEE J. Biomed. Health Informatics 18, 1804–1812. 10.1109/JBHI.2014.2329841 25014974

[B21] Kantak S. Jax S. Wittenberg G. (2017). Bimanual coordination: a missing piece of arm rehabilitation after stroke. Restor. Neurol. Neurosci. 35, 347–364. 10.3233/RNN-170737 28697575

[B22] Lang C. E. Waddell K. J. Klaesner J. W. Bland M. D. (2017). A method for quantifying upper limb performance in daily life using accelerometers. J. Visual. Exp. 2017:e55673. 10.3791/55673 28518079 PMC5565027

[B23] Laput G. Harrison C. (2019). Sensing fine-grained hand activity with smartwatches, in Proceedings of the 2019 CHI Conference on Human Factors in Computing Systems (Glasgow), 1–13. 10.1145/3290605.3300568

[B24] Lemmens R. J. Timmermans A. A. Janssen-Potten Y. J. Smeets R. J. Seelen H. A. (2012). Valid and reliable instruments for arm-hand assessment at ICF activity level in persons with hemiplegia: a systematic review. BMC Neurol. 12:21. 10.1186/1471-2377-12-21 22498041 PMC3352056

[B25] Leuenberger K. Gonzenbach R. Wachter S. Luft A. Gassert R. (2017). A method to qualitatively assess arm use in stroke survivors in the home environment. Med. Biol. Eng. Comput. 55, 141–150. 10.1007/s11517-016-1496-7 27106757 PMC5222943

[B26] Levin M. F. (1996). Interjoint coordination during pointing movements is disrupted in spastic hemiparesis. Brain 119, 281–293. 10.1093/brain/119.1.281 8624689

[B27] Levin M. F. Kleim J. A. Wolf S. L. (2009). What do motor “recovery” and “compensation” mean in patients following stroke? Neurorehabil. Neural Repair 23, 313–319. 10.1177/1545968308328727 19118128

[B28] Levin M. F. Michaelsen S. M. Cirstea C. M. Roby-Brami A. (2002). Use of the trunk for reaching targets placed within and beyond the reach in adult hemiparesis. Exp. Brain Res. 143, 171–180. 10.1007/s00221-001-0976-6 11880893

[B29] Lorussi F. Carbonaro N. De Rossi D. Paradiso R. Veltink P. Tognetti A. (2016). Wearable textile platform for assessing stroke patient treatment in daily life conditions. Front. Bioeng. Biotechnol. 4:28. 10.3389/fbioe.2016.00028 27047939 PMC4803737

[B30] Lum P. S. Shu L. Bochniewicz E. M. Tran T. Chang L.-C. Barth J.. (2020). Improving accelerometry-based measurement of functional use of the upper extremity after stroke: machine learning versus counts threshold method. Neurorehabil. Neural Repair 34, 1078–1087. 10.1177/1545968320962483 33150830 PMC7704838

[B31] Maceira-Elvira P. Popa T. Schmid A.-C. Hummel F. C. (2019). Wearable technology in stroke rehabilitation: towards improved diagnosis and treatment of upper-limb motor impairment. J. NeuroEng. Rehabil. 16:142. 10.1186/s12984-019-0612-y 31744553 PMC6862815

[B32] Malešević N. Wang C. F. Rich K. Antfolk C. (2019). Fall prevention for elderly people using radar sensor: feasibility study, in RESNA Annual Conference 2019 (Toronto, ON).

[B33] Mallinson T. Hammel J. (2010). Measurement of participation: intersecting person, task, and environment. Arch. Phys. Med. Rehabil. 91(9 Suppl.), S29–S33. 10.1016/j.apmr.2010.04.027 20801276

[B34] Mansur P. H. G. Cury L. K. P. Andrade A. O. Pereira A. A. Miotto G. A. A. Soares A. B.. (2007). A review on techniques for tremor recording and quantification. Crit. Rev. Biomed. Eng. 35, 343–362. 10.1615/CritRevBiomedEng.v35.i5.10 19392642

[B35] McLeod A. Bochniewicz E. M. Lum P. S. Holley R. J. Emmer G. Dromerick A. W. (2016). Using wearable sensors and machine learning models to separate functional upper extremity use from walking-associated arm movements. Arch. Phys. Med. Rehabil. 97, 224–231. 10.1016/j.apmr.2015.08.435 26435302

[B36] Melendez-Calderon A. Shirota C. Balasubramanian S. (2021). Estimating movement smoothness from inertial measurement units. Front. Bioeng. Biotechnol. 8:1507. 10.3389/fbioe.2020.558771 33520949 PMC7841375

[B37] Nguyen T.-H.-C. Nebel J.-C. Florez-Revuelta F. (2016). Recognition of activities of daily living with egocentric vision: a review. Sensors 16:72. 10.3390/s16010072 26751452 PMC4732105

[B38] Nweke H. F. Teh Y. W. Al-Garadi M. A. Alo U. R. (2018). Deep learning algorithms for human activity recognition using mobile and wearable sensor networks: state of the art and research challenges. Expert Syst. Appl. 105, 233–261. 10.1016/j.eswa.2018.03.056

[B39] Ploderer B. Fong J. Klaic M. Nair S. Vetere F. Lizama L. E. C.. (2016). How therapists use visualizations of upper limb movement information from stroke patients: a qualitative study with simulated information. JMIR Rehabil. Assist. Technol. 3:e9. 10.2196/rehab.6182 28582257 PMC5454558

[B40] Rand D. Eng J. J. (2012). Disparity between functional recovery and daily use of the upper and lower extremities during subacute stroke rehabilitation. Neurorehabil. Neural Repair 26, 76–84. 10.1177/1545968311408918 21693771 PMC3233607

[B41] Schambra H. M. Parnandi A. Pandit N. G. Uddin J. Wirtanen A. Nilsen D. M. (2019). A taxonomy of functional upper extremity motion. Front. Neurol. 10:857. 10.3389/fneur.2019.00857 31481922 PMC6710387

[B42] Subash T. David A. Skm V. Balasubramanian S. (2020). Comparison of wearable sensor 18 based algorithms for upper limb activity detection, in International Conference on NeuroRehabilitation (Springer).

[B43] Taub E. Uswatte G. Mark V. Morris D. (2006). The learned nonuse phenomenon: implications for rehabilitation. Eura Medicophys. 42, 241–255. 17039223

[B44] Tsai M.-F. Wang R. H. Zariffa J. (2020). Generalizability of hand-object interaction detection in egocentric video across populations with hand impairment, in 2020 42nd Annual International Conference of the IEEE Engineering in Medicine & Biology Society (EMBC), 3228–3231. 10.1109/EMBC44109.2020.9176154 33018692

[B45] Tsai M.-F. Wang R. H. Zariffa J. (2021). Identifying hand use and hand roles after stroke using egocentric video. IEEE J. Transl. Eng. Health Med. 9:2100510. 10.1109/JTEHM.2021.3072347 33889453 PMC8055062

[B46] Uswatte G. Giuliani C. Winstein C. Zeringue A. Hobbs L. Wolf S. L. (2006a). Validity of accelerometry for monitoring real-world arm activity in patients with subacute stroke: evidence from the extremity constraint-induced therapy evaluation trial. Arch. Phys. Med. Rehabil. 87, 1340–1345. 10.1016/j.apmr.2006.06.006 17023243

[B47] Uswatte G. Taub E. Morris D. Light K. Thompson P. (2006b). The motor activity log-28: assessing daily use of the hemiparetic arm after stroke. Neurology 67, 1189–1194. 10.1212/01.wnl.0000238164.90657.c2 17030751

[B48] Van Meulen F. B. Klaassen B. Held J. Reenalda J. Buurke J. H. Van Beijnum B.-J. F.. (2016). Objective evaluation of the quality of movement in daily life after stroke. Front. Bioeng. Biotechnol. 3:210. 10.3389/fbioe.2015.00210 26793705 PMC4710748

[B49] von Ziegler L. Sturman O. Bohacek J. (2020). Big behavior: challenges and opportunities in a new era of deep behavior profiling. Neuropsychopharmacology 46, 33–44. 10.1038/s41386-020-0751-7 32599604 PMC7688651

